# Impaired RIPK1 ubiquitination sensitizes mice to TNF toxicity and inflammatory cell death

**DOI:** 10.1038/s41418-020-00629-3

**Published:** 2020-09-30

**Authors:** Matthias Kist, László G. Kőműves, Tatiana Goncharov, Debra L. Dugger, Charles Yu, Merone Roose-Girma, Kim Newton, Joshua D. Webster, Domagoj Vucic

**Affiliations:** 1 Departments of Early Discovery Biochemistry, Genentech, 1 DNA Way, South San Francisco, CA 94080 USA; 2 Pathology, Genentech, 1 DNA Way, South San Francisco, CA 94080 USA; 3 Physiological Chemistry, Genentech, 1 DNA Way, South San Francisco, CA 94080 USA; 4 Molecular Biology, Genentech, 1 DNA Way, South San Francisco, CA 94080 USA

**Keywords:** Acute inflammation, Chronic inflammation

## Abstract

Receptor-interacting protein 1 (RIP1; RIPK1) is a key regulator of multiple signaling pathways that mediate inflammatory responses and cell death. TNF-TNFR1 triggered signaling complex formation, subsequent NF-κB and MAPK activation and induction of cell death involve RIPK1 ubiquitination at several lysine residues including Lys376 and Lys115. Here we show that mutating the ubiquitination site K376 of RIPK1 (K376R) in mice activates cell death resulting in embryonic lethality. In contrast to *Ripk1*^*K376R/K376R*^ mice, *Ripk1*^*K115R/K115R*^ mice reached adulthood and showed slightly higher responsiveness to TNF-induced death. Cell death observed in *Ripk1*^*K376R/K376R*^ embryos relied on RIPK1 kinase activity as administration of RIPK1 inhibitor GNE684 to pregnant heterozygous mice effectively blocked cell death and prolonged survival. Embryonic lethality of *Ripk1*^*K376R/K376R*^ mice was prevented by the loss of TNFR1, or by simultaneous deletion of caspase-8 and RIPK3. Interestingly, elimination of the wild-type allele from adult *Ripk1*^*K376R/cko*^ mice was tolerated. However, adult *Ripk1*^*K376R/cko*^ mice were exquisitely sensitive to TNF-induced hypothermia and associated lethality. Absence of the K376 ubiquitination site diminished K11-linked, K63-linked, and linear ubiquitination of RIPK1, and promoted the assembly of death-inducing cellular complexes, suggesting that multiple ubiquitin linkages contribute to the stability of the RIPK1 signaling complex that stimulates NF-κB and MAPK activation. In contrast, mutating K115 did not affect RIPK1 ubiquitination or TNF stimulated NF-κB and MAPK signaling. Overall, our data indicate that selective impairment of RIPK1 ubiquitination can lower the threshold for RIPK1 activation by TNF resulting in cell death and embryonic lethality.

## Introduction

RIPK1 (Receptor interacting protein kinase 1, RIP1) plays a central role in mediating signaling by TNF (Tumor necrosis factor) [1, 2]. Besides triggering proinflammatory and survival signals mediated by NF-κB (nuclear factor –κB) and MAPK (mitogen-activated protein kinase), TNF can also promote cell death (apoptosis and necroptosis) [3]. Phosphorylation [4–7], proteolytic cleavage [8–10], and ubiquitination of RIPK1 modulates signaling outcomes [11]. Lysine 377 (K377) of human RIPK1 was shown to be a critical residue for complex I formation [12, 13]. A RIPK1(K377R) mutant showed reduced ubiquitination and impaired recruitment of RIPK1 to the TNFR1 receptor complex, leading to decreased NF-κB activation [12, 14]. Another reported ubiquitination site on RIPK1, K115, is ubiquitinated during TNF-induced necroptotic cell death [15–17].

TNFR1 (TNF receptor 1) trimerization induced by TNF binding results in recruitment of TRAF2 (TNF receptor-associated factor 2), TRADD (TNF receptor type 1 associated death domain protein), RIPK1 and c-IAP1 and 2 (cellular inhibitor of apoptosis 1 and 2). The E3 ligases c-IAP1/2 then ubiquitinate several proteins in the complex, including themselves and RIPK1, with K11-, K48- and K63-linked chains [18–21]. K63-linked ubiquitin chains conjugated on c-IAP1/2 enable the binding of LUBAC (linear ubiquitin chain assembly complex), which subsequently adds linear ubiquitin chains [22]. NEMO (NF-kappa-B essential modulator) binds to linear chains, which enables recruitment of IKKα/β (inhibitor of nuclear factor kappa-B kinaseα/β) and NF-κB activation [23]. K63-linked ubiquitin-binding proteins TAB2/3 (TAK1-binding proteins 2 and 3) bring TAK1 (transforming growth factor beta-activated kinase 1) to the complex [22, 24], which also contributes to activation of NF-κB and MAPK signaling [25].

Persistent pathway activation and deubiquitination of RIPK1 can induce complex II formation. Complex II is a cytosolic complex consisting of caspase-8, FADD (Fas-associated protein with death domain), FLIP (FLICE inhibitory protein) and RIPK1 [20, 26]. This complex can activate caspase-3 and -7 resulting in apoptosis, but will recruit RIPK3 to form complex IIb if caspases are inhibited or absent, leading to necroptosis [1, 2]. RIPK1 is activated by autophosphorylation and becomes ubiquitinated at the same time [15, 16]. Subsequently, active RIPK1 binds to RIPK3, which leads to RIPK3 autophosphorylation and phosphorylation of pseudokinase MLKL (mixed lineage kinase domain like) [27–29]. Phosphorylated MLKL oligomerizes and translocates to the plasma membrane inducing membrane perturbations that result in cell lysis [30].

RIPK1 has been implicated in numerous inflammatory and neurodegenerative pathologies in animal disease models, and by demonstration of RIPK1 pathway activation in patients [31, 32]. Multiple studies have shown that genetic inactivation or chemical inhibition of RIPK1 kinase activity is protective in animal disease models of gut and skin inflammation, rheumatoid arthritis, Alzheimer’s disease, multiple sclerosis and Parkinson’s disease, to name a few [31, 32]. RIPK1 inhibition is also protective in acute disease models such as the TNF-induced systemic inflammatory response syndrome (SIRS) model [31, 33]. All these studies suggest that RIPK1 is an attractive drug target in inflammatory and neurodegenerative diseases [32, 34].

While it is clear that the kinase activity of RIPK1 is dispensable for organismal homeostasis or reproduction, the physiological importance of RIPK1 ubiquitination is less clear. To assess the biological relevance of pro-survival and pro-cell death ubiquitination of RIPK1, we generated knock-in mice where K376 or K115 was mutated to arginine. RIPK1(K376R) reduced K11-linked, K63-linked, and linear RIPK1 ubiquitination, and promoted the assembly of death-inducing cellular complexes. Apoptosis in embryos caused lethality and was dependent on the catalytic activity of RIPK1, TNFR1, and caspase-8/RIPK3. RIPK1(K115R) was not lethal, but moderately enhanced responsiveness to TNF-induced death. Together, our data suggest that selective impairment of RIPK1 ubiquitination promotes activation of RIPK1, cell death, and embryonic lethality.

## Methods

### Mice

The Genentech institutional animal care and use committee responsible for ethical compliance approved all animal protocols. *Tnfr1*^*−/−*^, *Casp8*^*+/−*^, *Ripk3*^−*/−*^ and *Rosa26-Cre.ER*^*T2*^ mice were described before [33, 35, 36]. *Ripk1*^*K115R*^ knockin mice were generated by CRISPR/Cas9 technology [37, 38]. The guide target sequence was: 5’ GAA AGG AAG GAT AAT CGT GG 3’ with protospacer adaptive motif (PAM): AGG and an oligonucleotide donor 5’CTT CAG GTC CTT GTG TAT CAC ACC TTT GTC ATG TAA GTA GCA CAT GCC TTC TAT tgc ttc tac aat TAT CCT TCC tcg CAA TGA AAG TGG GAC ATC TAT CTG GAA TAA CAC ATT AAG TCT ATG AAG TGA AGA GGC AAT CTA ACA GGC AAG AGC 3’ (Integrated DNA technologies) were used. Cas9 mRNA (Thermo Fisher; A29378), sgRNA (Synthego) and oligonucleotide template were used to modify zygotes. After zygote microinjection and embryo transfer, genomic DNA was prepared from tail tip biopsies of potential G0 founders. G0 mosaic founders were analyzed for the top 15 off-target loci per sgRNA (obtained by the CRISPR design tool from Benchling). Founders without mutations were selected for mating with wild type C57BL/6N mice for germline transmission of the gene-edited chromosome.

The same approach was chosen for *Ripk1*^*K376R*^ knockin strain. The guide target sequence was: 5’ CGAGAATGATCGCAGTGTGC 3’; PAM: AGG and the oligonucleotide donor (5’ATT CTG CCT TGG CTG CGG TTT TGT CTG TTT CTC TGC AAA TAT TCC AAA AGC ATG ATA GCT GGC TTC CTC TTG CAG tcg tgc CTG tac ACT gcg atc ATT CTC GTC CTG TGG GTA CTC TGG GGA GGA AGA AAA CCA GGA CTC CTC CAC AGG ACC 3’) was used.

All alleles were maintained on a C57BL/6N genetic background. *Ripk1*^*K115R*^ genotyping primers (5’-CTA TAG GCC CTG GGG TAA A-3’and 5’- CTT CTA TTG CTT CTA CAA TTA TCC-3’) amplified a 242 bp DNA fragment. Ripk1WT genotyping primers (5’-CTA TAG GCC CTG GGG TAA A-3’ and 5’-CCT CCA CGA TTA TCC TTC CT-3’) amplified a 233 bp DNA fragment.

*Ripk1*^*K376R*^ genotyping primers (5’-CAG TGT GCA GGC TAA GCT-3’ and 5’-CAC TGC AAT TCC ACG ACT C-3’) amplify a 228 bp DNA fragment. *RIPK1*^*WT*^ genotyping primers (5’-GTA CAG GCA CGA CTG C-3’ and 5’-CAC TGC AATT CCA CGA CTC-3’) amplified a 232 bp DNA fragment.

Taconic (Germany) generated *Ripk1*^*cko/+*^ mice using C57BL/6NTac ES cells (schematic Supplementary Fig. S5A). The Ripk1 floxed region corresponds to genomic position (GRCm38/mm10 assembly): chr13:34,009,835-34,010,565 containing exon 2. Mice were maintained on a C57BL/6N background.

Genotyping for the *Ripk1*^*cko*^ allele was performed with 3 primers resulting in the different amplicons based on genotype (Primer 1: 5’-TAC GAG GAA GAC ATC ACT GAA GAC-3’, Primer 2: 5’-AAC TAG CCT GAG GAG AAG AGA AG-3’ and Primer 3: 5’-AGC ATA AGA CAC AGA CCC TAA CA-3’). The following amplicon lengths were expected based on genotype: WT 188 bp, loxp: 248 bp and recombined KO: 329 bp.

For timed pregnancies, the day of vaginal plug detection was set as embryonic day 0.5 (E0.5). Pregnancies were confirmed by ultrasound or by weight gain of the dams. RIPK1 kinase inhibitor GNE684 was dosed by oral gavage twice day at 10 mg/ml in 10% DMSO + MCT. The mice were dosed with 50 mg/kg GNE684.

Systemic inflammatory response syndrome (SIRS) was induced by intravenous (iv) injection of 500 μg of mouse TNF (Genentech) [39] per kg body weight. Calculations to determine group sizes were not performed, mice were grouped according to genotypes and the studies were unblinded. Body temperature was monitored using a rectal probe and a digital thermometer. Mice were euthanized if their body temperature was below 25 °C or if severely lethargic.

The *Rosa26-Cre.ER*^*T2*^ allele was maintained heterozygous. Nuclear translocation was induced by intraperitoneal injection of tamoxifen (80 mg/kg body weight) for three consecutive days. Tamoxifen (Sigma-Aldrich) was solubilized in sunflower seed oil (Sigma-Aldrich). Days of experiments or analysis are indicated in the Figure legends. Mice were excluded from experiments if they had clinical observations or fight wounds. No mice or samples were excluded.

### Histology and Immunohistochemistry (IHC)

For Histology, mouse tissues were formalin fixed, paraffin embedded, sectioned and stained with Hematoxylin and eosin stain (H&E). Formalin-fixed paraffin-embedded tissue sections were stained with 0.05 μg/ml rabbit anti-cleaved caspase-3 (Asp175) (CST, 9661) or 5 μg/ml rabbit anti-phospho-RIPK3 Thr231, Ser232 (Genentech GEN135-35-9). IHC for cleaved caspase-3 and pRIPK3 was performed on the Ventana Discovery XT platform with CC1 standard antigen retrieval (Ventana). Cleaved caspase-3 was detected with the Ventana OmniMap detection system and DAB chromogen (Ventana). The pRIPK3 signal was amplified with Ventana HQ Amplification and detected with HQ Discovery detection systems and DAB chromogen.

### Cells and reagents

Primary MEFs were isolated from E12.5 (K376R) and E14.5 (K115R) embryos (excluding head) using the Pierce Mouse Embryonic Fibroblast Isolation Kit (88279) following the manufactures instructions. The cells were cultured in Dulbecco’s modified Eagle medium High Glucose medium containing 10% fetal bovine serum, 2 mM GlutaMAX (Gibco), 1x non-essential amino acids (Gibco), 100 U/ml Penicillin, 100 μg/ml Streptomycin (Gibco). The tissue culture plates were coated with 0.1% gelatin in 1x PBS. *Ripk1*^*K376R/K376R*^ and littermate *Ripk1*^*WT/WT*^ primary MEFs were immortalized using retroviral vector pWZL-hygro-E1A and pBABE-neo-H-ras G12V expression constructs.

BMDMs were extracted from femurs and tibiae of adult mice. Cells were cultured for 6 days in DMEM High Glucose supplemented with 10% heat-inactivated fetal bovine serum, 2 mM GlutaMAX (Gibco), 1x non-essential amino acids (Gibco), 100 U/ml Penicillin and 100 μg/ml Streptomycin (Gibco), 50 μM β-mercaptoehtanol, 10 mM HEPES pH 7.4 and 20% L929 pre-conditioned media. The cells were cultured on non-treated plates. For induction of *Rosa26-Cre.ER*^*T2*^ *Ripk1*^*cko/K376R*^ and *Ripk1*^*cko/WT*^ BMDMs were treated with 30 nM 4-OH-tamoxifen (Sigma-Aldrich) for the differentiation.

Cell death was analyzed using Incucyte ZOOM and S3 (Essen BioSciences) using Sytox Green nucleic acid stain (Life technologies). 200 μg/ml digitonin (Sigma Aldrich) was used as a positive control to achieve complete cell lysis.

Cells were treated with 5, 100 or 1000 ng/ml murine TNF (Genentech) [39], 10 ng/ml LPS (Invivogen, tlrl-3pelps), 10 μg/ml Poly(I:C) (Invivogen, tlrl-picw) transfected with Lipofectamine 2000 (Invitrogen, 11667-030), 20 μM zVAD (ABclonal), indicated concentrations of BV6 (ref. [40] ), 5 μM GNE684 (ref. [31]) and 1 μg/ml murine FLAG-TNF (Enzo, ALX-522-009-C050).

### Western blotting and immunoprecipitation

Cells were lysed in 1% Triton X-100 (w/v), 25 mM Tris HCl pH 7.5, 150 mM NaCl, 1 mM EDTA, 10% glycerol, PhosSTOP phosphatase inhibitor [1x] (Roche) and complete protease inhibitor cocktail [1x] (Roche). For immunoprecipitations cells were lysed as described above with the addition of 10 mM N-ethylmaleimide (NEM, Sigma-Aldrich). Insoluble fraction was separated from the lysate by centrifugation at 20,817 xg.

Western blotting antibodies against: RIPK1 (BD, 610459), RIPK1(Genentech, 10C7.3.1, 1 μg/ml), pRIPK1 Ser166; Thr169 (Genentech, GEN175-DP-A1, 1 μg/ml), RIPK3 (Genentech, 1G6.1.4, 1 μg/ml), pRIPK3 Thr231; Ser232 (Genentech, GEN135-35-9, 1 μg/ml), MLKL (Milipore, MABC604), pMLKL (abcam, ab196436), FADD (Milipore, 05-486), pIκBα (CST, 2859), IκBα (CST, 9242), p-p65 (CST, 3033), p65 (CST, 8242), pJNK (BD, 612541), JNK (BD, 554286), p-p38 (CST, 9211), p38 (CST, 8690), TRADD (Genentech, GN21-3, 1 μg/ml), HOIP (Genentech, 11D6H2G5, 1 μg/ml), SHARPIN (Proteintech, 14626-1-AP), NEMO (abcam, ab178872), caspase-3 (CST, 9662), cleaved capsase-3 (CST, 9661), caspase-8 [1G12] (Enzo, ALX-804-447-C100), FLIP (CST, 56343), p-ERK (CST, 4370), ERK (CST, 4695), c-IAP1 (Enzo, ALX-803-335), pIKKα/β (CST, 2697), IKKβ (CST, 8943), GAPDH (CST, 2118, 1:5000), HDAC2 (CST, 5113), HSP90 (CST, 4874, 1:2000). If not otherwise indicated the antibodies were diluted 1:1000 for detection.

IP was performed with anti-RIPK1 antibody (BD, 610459) or anti-TNFR1 antibody (RnD, AF-425). Complexes were purified using magnetic A/G beads (Pierce) and eluted with 1x sample buffer. For cells treated with FLAG-TNF immunoprecipitation was performed with magnetic FLAG-M2 beads (Sigma-Aldrich, M8823).

Ubiquitin chain-specific IPs were performed by lysing the cells in 6 M urea containing buffer (20 mM Tris–HCl (pH 7.5), 135 mM NaCl, 1.5 mM MgCl_2_, 1 mM EGTA, 1% (w/v) Triton X-100, 10 mM N-ethylmaleimide (NEM, Sigma Aldrich) and Halt Protease and Phosphatase Inhibitor Cocktail (Thermo Scientific) as recently described [41]. Ub-IP was performed with linkage-specific antibodies: anti-K11-Ub (Genentech, 2A3/2E6), anti-K63-Ub (Genentech, APU3.A8), anti-Lin-Ub (Genentech, 1F11/3F5/Y102L). Detection was performed with anti-RIPK1 antibody (BD, 610459).

### Immunofluorescence

Yolk sacs were stained as described in Newton et al. [33]. Fixed tissues were stained with rat anti-PECAM-1 (BD, 550274) and rabbit anti-cleaved caspase-3 (CST, 9661). The following secondary antibodies were used: donkey anti-rabbit Cy3 (Jackson ImmunoResearch, 711-165-152) and donkey anti-rat Cy5 (Jackson ImmunoResearch, 712-175-153). Processed yolk sacs were mounted using ProLong Gold Antifade Mountant with DAPI (Invitrogen, P36392) and images were acquired using a LEICA SPE upright confocal microscope with 20x objective. On average about 50 optical sections were collected, representing about 50 μm deep volume, with 1.19 μm step size. The images shown are maximum intensity projections.

BMDMs were seeded at d5 on 4 chamber tissue culture treated glass slides (Falcon, 354104) and treated the following day. The cells were fixed in 4% PFA for 30 min at room temperature. Permeabilized in 0.25% Triton X-100 (w/v) and blocked in 5% BSA (w/v) and 0.05% Triton X-100 (w/v). Staining was performed with p65 (CST, 8242) for 2 h at room temperature followed by labeling with donkey anti-rabbit Cy3 (Jackson ImmunoResearch, 711-165-152) and 1:2000 Hoechst (H3569). The cells were mounted with ProLong Glass Antifade Mountant (P36980). Images were acquired using a LEICA SP8 inverted microscope with a 40x objective.

### Quantitative reverse transcription PCR (RT-qPCR)

Total RNA was extracted from cells or tissues samples using the RNeasy plus mini kit following manufactures instructions. cDNA was generated using the SuperScript IV VILO Master Mix. qRT-PCR was performed with pre-spotted and costume assembled TaqMan assay plates (Thermo Fisher Scientific) and TaqMan Fast Advanced Master Mix (Thermo Fisher Scientific, 4444557). Analysis was performed with the QuantStudio 7 and normalized to expression of *Ppia* (Ppia-Mm02342430_g1). The following assays were used: Ppia-Mm02342430_g1, Tnfaip3-Mm00437121_m1, Cxcl1-Mm04207460_m1, Cxcl2-Mm00436450_m1, Ccl2-Mm00441242_m1, Il1b-Mm00434228_m1, Tnf-Mm00443258_m1, Relb-Mm00485664_m1, Bcl3-Mm00504306_m1 (all Thermo Fisher Scientific).

### Cytokine and chemokine detection

Sera of adult mice or cell supernatants were analyzed by Luminex Panel (BioRad). The following cytokines and chemokines were analyzed from cell supernatants: MIP1α (R&D Systems, MMA00), IL-6 (R&D Systems, D6050), G-CSF (R&D Systems, MCS00).

### Statistical analysis

The number of independent experiments performed is indicated in the figure legends (at least two). The variance was assumed to be similar between the compared groups and that groups have normal distribution. The statistical significance was analyzed by the indicated tests. One-way ANOVA, Two-way ANOVA and Mantel–Cox (log rank) were performed using the GraphPad Prism software.

## Results

### RIPK1 ubiquitination at lysines 376 and 115 differentially regulates survival during embryogenesis

**Fig. 1:**
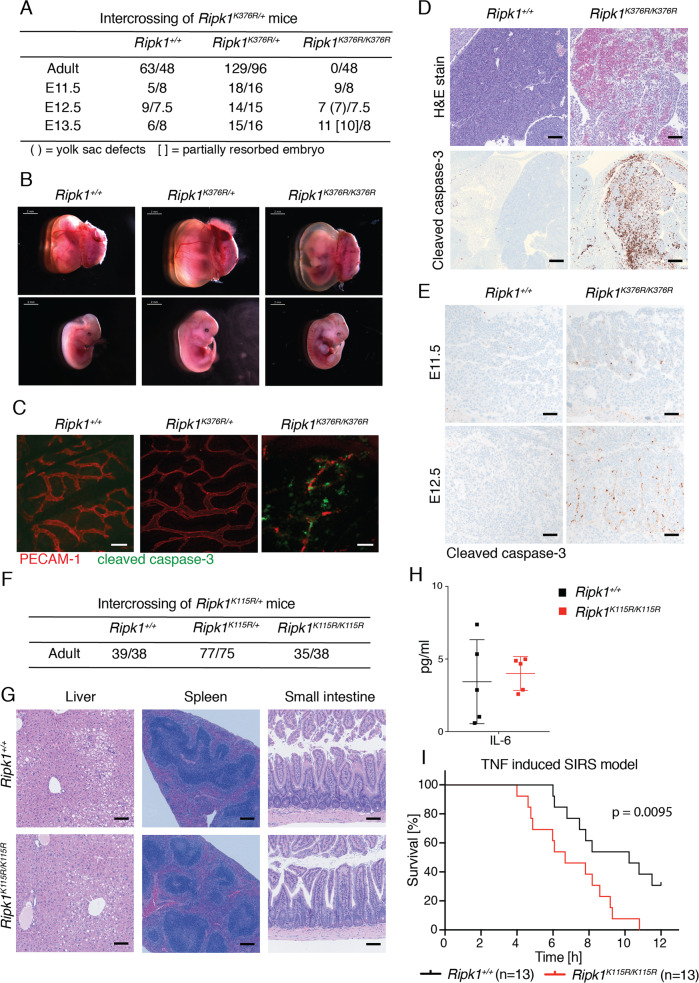
RIPK1 K376R mutation cause embryonic lethality, while RIPK1 K115R mutation does not affect survival. **a** Numbers of offspring at different embryonic days and adult age from intercrossing *Ripk1*^*K376R/+*^ mice. **b** Representative embryos of the *Ripk1*^*K376R/+*^ intercross at E12.5. Scale bars on the top left of each image indicate 2 mm. **c** Yolk sac of *Ripk1*^*+/+*^, *Ripk1*^*K376R/+*^ and *Ripk1*^*K376R/K376R*^ intercross labeled for cleaved caspase-3 [green] and PECAM-1 [red] [scale bar 50 μm]. **d** Hematoxylin and eosin staining [upper panels, scale bar 100 μm] and IHC for cleaved caspase-3 [lower panels, scale bar 200 μm] of *Ripk1*^*+/+*^ and *Ripk1*^*K376R/K376R*^ embryos. **e** IHC labeling for cleaved caspase-3 in the placenta labyrinths of *Ripk1*^*+/+*^ and *Ripk1*^*K376R/K376R*^ embryos [scale bar 100 μm]. **f** Numbers of offspring from intercrossing *Ripk1*^*K115R/+*^ mice. **g** Histology of liver, spleen and small intestines of 12–15 months aged *Ripk1*^*+/+*^ and *Ripk1*^*K115R/K115R*^ mice [scale bar 100 μm liver and small intestine, 200 μm spleen]. **h** Serum cytokine levels of IL-6 in *Ripk1*^*+/+*^ [*n* = 5] and *Ripk1*^*K115R/K115R*^ [*n* = 5] 12–15 months old. **i** Survival of *Ripk1*^*+/+*^ in black [*n* = 13 males] and *Ripk1*^*K115R/K115R*^ in red [*n* = 13 males] mice in TNF-induced SIRS model with 500 μg/kg TNF injected iv. Difference between the two groups by Mantel–Cox test: *p* = 0.0095.

Lysines 376 and 115 of RIPK1 are ubiquitinated in response to TNFR1 signaling [12, 14, 15]. Ubiquitination of RIPK1 at lysine 376 occurs in the TNFR1-associated complex I and is linked to activation of NF-κB and MAPK signaling [12, 14]. To analyze the role of K376 under physiological conditions, RIPK1(K376R) mutant knock-in (KI) mice were generated using CRISPR technology (see Methods). Interbreeding of *Ripk1*^*K376R/+*^ animals did not produce viable *Ripk1*^*K376R/K376R*^ (hereafter termed K376R) mice (Fig. 1a). Analysis of timed pregnancies indicated that K376R embryos were grossly normal up until embryonic day 11.5 (E11.5). At E12.5, however, K376R embryos suffered from macroscopic devascularization of the yolk sac and, by E13.5, showed signs of resorption (Figs. 1b and S1A). Immunolabeling of yolk sacs of wild-type (WT), heterozygous and homozygous K376R embryos at E12.5 for PECAM-1 (an endothelial cell marker) and cleaved caspase-3 (a cell death marker) confirmed the vasculature breakdown and endothelial cell death (Fig. 1c). Cell death indicated by cleaved caspase-3 was also prominent in the liver of K376R embryos, and coincided with visible tissue breakdown (Fig. 1d). Cells containing cleaved caspase-3 were seen in K376R embryonic liver as early as E11.5 (Fig. S1B). Intriguingly, both cleaved caspase-3 and phosphorylated RIPK3 were detected in the K376R placenta, suggesting activation of apoptotic and necroptotic cell death pathways (Figs. 1e and S1C, D).

K115 ubiquitination of RIPK1 has been linked to necroptotic cell death signaling [15, 17]. To investigate the role of this ubiquitination site in vivo, CRISPR RIPK1 K115R KI mice were generated (see Methods). *Ripk1*^*K115R/K115R*^ (K115R) mice were born at normal Mendelian frequencies (Fig. 1f). Aging for 15 months did not result in any genotype-related differences between WT and K115R mice, but comparable age-related findings including neoplasia were identified in both genotypes (Fig. 1g). In line with these findings, serum IL-6 levels were not different between WT and K115R animals (Fig. 1h). When K115R mice were challenged by intravenous injection of TNF (500 μg/kg body weight) in a TNF-induced systemic inflammatory response syndrome (SIRS) model, they exhibited a significantly higher morbidity (Mantel–Cox test *p* = 0.0095) (Fig. 1i) and a more pronounced drop in body temperature than WT littermates (Fig. S2A). K115R animals had slightly but not significantly elevated serum levels of IL-6 after 4 h (*p* = 0.25), whereas levels of other cytokines and chemokines such as RANTES, KC, and IFNγ were not altered (Fig. S2B). While RIPK1 K115R BMDMs (bone marrow-derived macrophages) exhibited normal ubiquitination of RIPK1 and normal NF-κΒ and MAPK signaling in response to TNF, the cells showed enhanced necroptosis signaling in response to TNF and zVAD (Fig. S2C-G). Necroptosis mediated by LPS and zVAD, or poly(I:C) and zVAD, was also slightly enhanced (Fig. S2G). Based on these results, RIPK1 ubiquitination at K115 seems to play a minor role in TNF-induced cell death signaling in vitro or in vivo.

### Inhibition of RIPK1 kinase activity blocks cell death and delays lethality in RIPK1(K376R) embryos

**Fig. 2:**
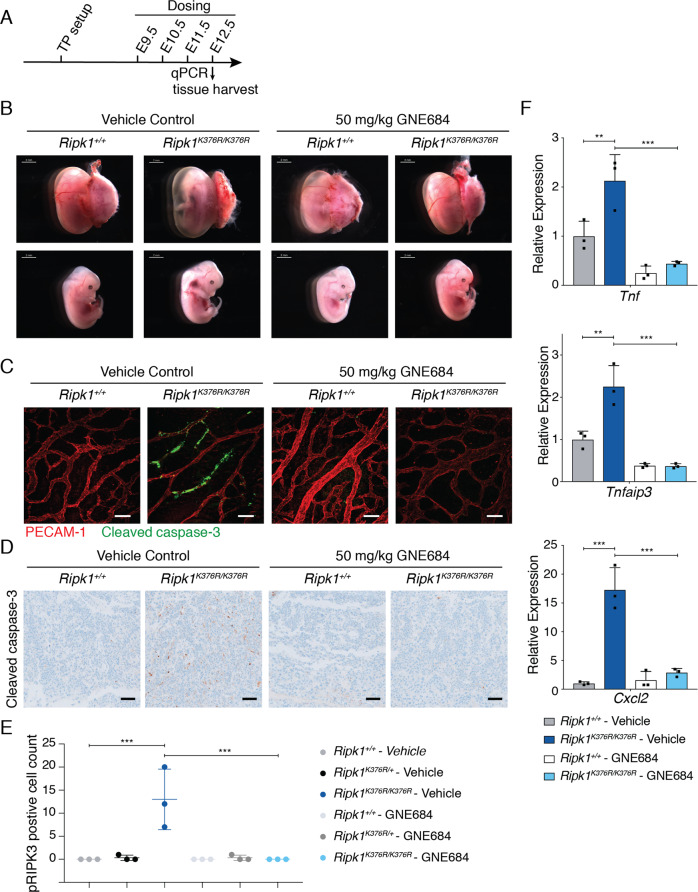
Inhibition of RIPK1 kinase activity blocks cell death and delays lethality in *Ripk1*^*K376R/K376R*^ embryos. **a** GNE684 dosing schematic. Pregnant females were doses by oral gavage twice a day with vehicle control or 50 mg/kg GNE684. **b** Representative embryos of the *Ripk1*^*K376R/+*^ intercross dosed for 4 days. Scale bars on the top left of each image indicate 2 mm. **c** Yolk sac of treated and non-treated embryos of *Ripk1*^*K376R/+*^ intercross immunolabeled for cleaved caspase-3 [green] and PECAM-1 [red] [scale bar 50 μm]. **d** IHC for cleaved caspase-3 of the representative placentas [scale bar 100 μm]. **e** Quantification of IHC for pRIPK3 positive cells in placentas of *Ripk1*^*+/+*^ and *Ripk1*^*K376R/K376R*^ at E12.5. Representative images are provided in S1F. **f** RT-qPCR analysis of indicated cytokines and chemokines using E11.5 embryonic liver total RNA [*n* = 3] after dosing for three days with vehicle or 50 mg/kg GNE684. Data of three independent experiments are shown as mean with SD. *P* values, one-way ANOVA followed by Tukey’s multiple comparison test (** > 0.0021, *** > 0.0002).

To investigate the role of RIPK1 catalytic activity in the lethality of RIPK1 K376R embryos, we dosed pregnant females with the RIPK1 kinase inhibitor GNE684 (ref. [31]). Pregnant females were dosed by oral gavage twice daily (50 mg/kg BID) starting at E9.5 (Fig. 2a) and embryos were necropsied at E12.5. As expected, K376R embryos from pregnant females treated with vehicle alone showed devascularization and blood vessel breakdown (Fig. 2b). Interestingly, K376R embryos exposed to GNE684 showed a complete rescue of the macroscopic phenotype (Fig. 2b). Accordingly, vehicle-treated K376R embryos had endothelial cells containing cleaved caspase-3, whereas GNE684-treated K376R embryos did not (Fig. 2c). Placentas of GNE684-treated embryos also lacked detectable cleaved caspase-3 (Figs. 2d and S1E). pRIPK3 positive cells were significantly increased in placentas of vehicle-treated K376R embryos compared to WT or treated K376R placentas (Figs. 2e and S1F). Thus, the kinase activity of RIPK1 appears crucial for RIPK1(K376R) to induce cell death and embryonic lethality at E12.5.

We also analyzed levels of proinflammatory cytokines and chemokines in E11.5 livers by quantitative PCR analysis. Several proinflammatory cytokine and chemokine genes (*Tnf*, *Cxcl2, Cxcl1*, and *Cxcl2*), as well as the NF-κB target genes *TNFAIP3*, *Bcl3*, and *RelB* were expressed at higher levels in K376R liver when compared to WT livers (Figs. 2f and S1G). Expression of these genes in K376R embryos treated with GNE684 was comparable to that seen in WT embryos (Figs. 2f and S1G), suggesting that cell death drives proinflammatory gene expression.

### RIPK1(K376R) impairs TNF-induced NF-κB and MAPK signaling and promotes cell death complex formation

**Fig. 3:**
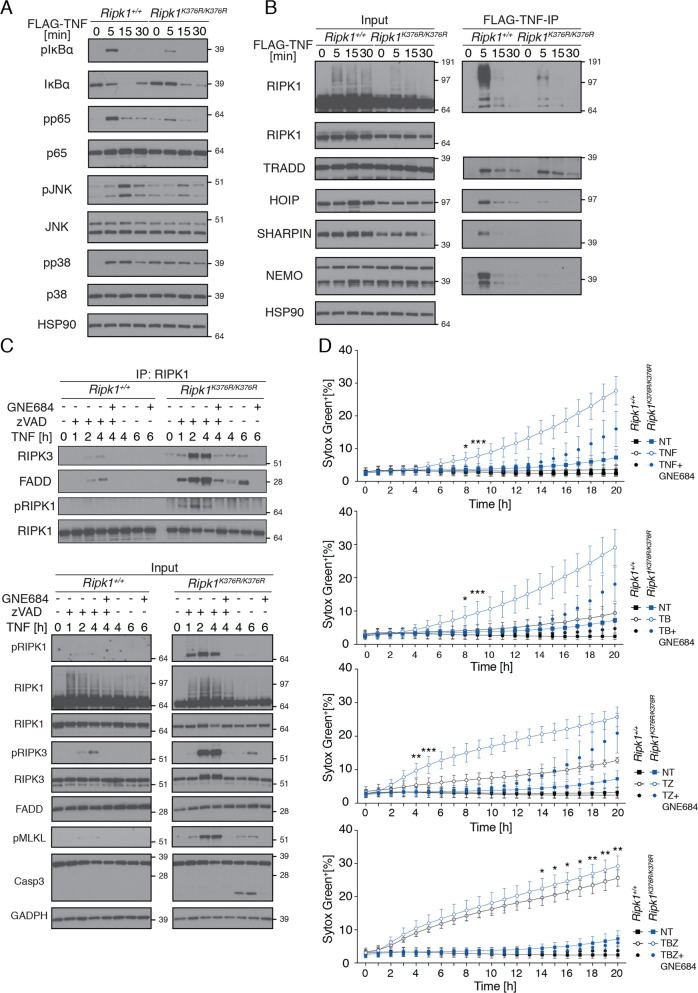
*Ripk1*^*K376R/K376R*^ reduces TNF-induced NF-κB and MAPK signaling and promotes cell death complex formation. **a** Western blot and **b** FLAG-TNF immunoprecipitation (IP) of the same experiment of *Ripk1*^*+/+*^ and *Ripk1*^*K376R/K376R*^ primary MEFs after treatment with 1 μg/ml FLAG-TNF for the indicates times, examined with indicated antibodies. **c** Western Blot of primary MEFs and IP after treatment with TNF [100 ng/ml], zVAD [20 μM] and GNE684 [5 μM] for the indicated time points. **d** Percentage of dead primary MEFs [Sytox Green positive cells] compared to positive control. Signal was measured every hour for 20 h in the Incucyte with the indicated treatments: TNF [T] 100 ng/ml, zVAD [Z] 20 μM, BV6 [B] 500 nM, GNE684 5 μM. The Mean value with SD for six independent experiments is shown. Asterisks indicate statistical analysis between treated WT and K376R samples. After 9 h for TNF and TB treatment and after 5 h for TZ, the significance did not change. *P* values, repeated measures two-way ANOVA followed by Tukey’s multiple comparison test (* > 0.0332, ** > 0.0021, *** > 0.0002).

To investigate the importance of RIPK1 lysine 376 for TNF-induced NF-κB and MAPK activation, as well as complex I formation, we treated primary MEFs (mouse embryo fibroblasts) derived from WT or K376R embryos with FLAG-TNF (Fig. 3a, b). RIPK1(K376R) decreased phosphorylation of IκBα, RelA/p65, JNK, and p38 when compared to RIPK1 WT (Fig. 3a). NF-κB activation was not affected in *Ripk1*^*K376R/+*^ primary MEFs compared to WT (Fig. S3A). Ubiquitination of RIPK1 and its recruitment to the TNFR1 receptor complex was also impaired in RIPK1(K376R) MEFs compared to WT MEFs (Fig. 3b). Accordingly, LUBAC components HOIP and SHARPIN as well as NEMO were only weakly recruited to the TNFR1 complex in K376R cells (Fig. 3b). TRADD recruitment to the complex was unchanged, which is expected given that TRADD is recruited via death domain interactions independently of RIPK1 ubiquitination (Fig. 3b). Subcellular fractionation analyses of RelA/p65 in immortalized MEFs treated with TNF confirmed the NF-κB activation defects observed in K376R primary MEFs. Translocation of phosphorylated p65 into the nucleus was impaired in K376R MEFs after TNF treatment (Fig. S3B). Interestingly, expression of select NF-κB target genes in primary MEFs after 4 h of TNF treatment was not greatly affected by RIPK1(K376R) (Fig. S3C, top row). Impaired TNF-induced gene expression was more apparent upon normalization to unstimulated cells of the same genotype (Fig. S3C, bottom row), possibly because of higher baseline gene expression in unstimulated K376R MEFs.

Defective recruitment of RIPK1(K376R) into complex I suggested that TNF-mediated cell death might be enhanced. Indeed, treatment with TNF for up to 6 h induced caspase-3 cleavage and autophosphorylation of RIPK3 in K376R MEFs, but not in WT MEFs (Fig. 3c). Signaling in response to TNF and zVAD (TZ) was also enhanced by RIPK1(K376R) based on increased phosphorylation of RIPK1, RIPK3, and MLKL, and more robust complex II formation in RIPK1 K376R MEFs when compared to WT MEFs (Fig. 3c). Again, we observed comparable signaling in *Ripk1*^*K376R/+*^ and WT MEFs (Fig. S3D). GNE684 blocked these signaling events, indicating their dependence on RIPK1 activation (Figs. 3c and S3E). Consistent with these data, RIPK1 K376R MEFs were more sensitive to either TNF- or TZ-induced cell death when compared to WT MEFs (Fig. 3d). Interestingly, addition of the IAP antagonist BV6 (ref. [40]) enhanced TNF- or TZ-induced cell death in WT, but not K376R MEFs (Fig. 3d). Indeed, WT and RIPK1 K376R MEFs exhibited comparable cell death in response to TNF, BV6, and zVAD (TBZ; Fig. 3d). Accordingly, TBZ induced comparable phosphorylation of RIPK1, RIPK3, and MLKL in WT and K376R MEFs (Fig. S3E). Thus, depletion of c-IAP1/2 by BV6 does not augment cell death in the absence of K376 RIPK1 ubiquitination site. K376R MEFs were also more sensitive to LPS and zVAD-induced cell death when compared to WT MEFs (Fig. S4A, B).

### Loss of TNFR1 or the combined loss of caspase-8 and RIPK3 prevents *Ripk1*^*K376R/K376R*^ embryonic lethality but not tissue inflammation

**Fig. 4:**
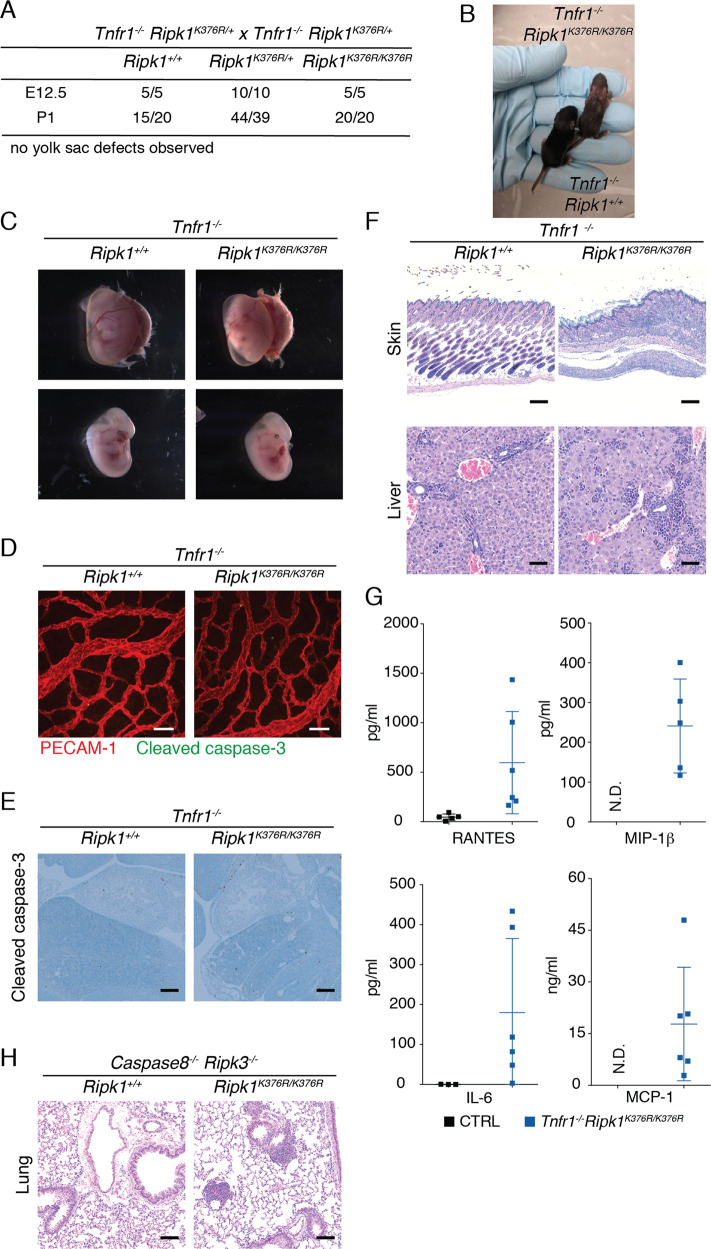
TNFR1 ablation rescues *Ripk1*^*K376R/K376R*^ embryonic lethality but not tissue inflammation. **a** Numbers of offspring at E12.5 and perinatal day 1 (P1) from intercrossing *Tnfr1*^−*/−*^ *Ripk1*^*K376R/+*^ mice. **b** Representative image of *Tnfr1*^*−/−*^ *Ripk1*^*+/+*^ and *Tnfr1*^*−/−*^ *Ripk1*^*K376R/K376R*^ pups at P7. **c** Representative embryos of the *Tnfr1*^*−/−*^ *Ripk1*^*K376R/+*^ intercross. Scale bars on the top left of each image indicate 2 mm. **d** Yolk sac of *Tnfr1*^*−/−*^ *Ripk1*^*+/+*^ and *Tnfr1*^*−/−*^ *Ripk1*^*K376R/K376R*^ intercross immunolabeled for with cleaved caspase-3 [green] and PECAM-1 [red] [scale bar 50 μm]. **e** IHC for cleaved caspase-3 of the representative embryos at E12.5. [scale bar 200 μm]. **f** Representative histology of skin [scale bar 200 μm] and liver [scale bar 50 μm] of *Tnfr1*^*−/−*^ *Ripk1*^*+/+*^ and *Tnfr1*^*−/−*^ *Ripk1*^*K376R/K376R*^. **g** Serum level cytokines and chemokines of MIP-1β, MCP-1, RANTES and IL-6 of *Tnfr1*^*−/−*^ *Ripk1*^*K376R/K376R*^ pups [*n* = 6] and littermate controls [*n* = 5]. Mean values with SD is plotted [N.D. not detected, 2 littermate control samples did not show detectable concentrations for IL-6]. **h** Lung of adult *Ripk1*^*K376R/K376R*^ *Casp8*^*−/−*^ *Ripk3*^*−/−*^ mice showed increased perivascular and peribronchiolar lymphoid aggregates compared to *Ripk1*^*+/+*^ *Casp8*^*−/−*^ *Ripk3*^*−/−*^ mice [scale bar 100 μm].

To examine if the lethality of K376R mice was dependent on TNF-induced cell death, *Ripk1*^*K376R/+*^ mice were crossed with either *Tnfr1*^*−/−*^ mice or *Casp8*^*−/−*^ *Ripk3*^*−/−*^ mice. *Ripk1*^*K376R/K376R*^ *Tnfr1*^*−/−*^ mice were born at Mendelian ratios (Fig. 4a), but were severely runted and had to be euthanized aged between 12 to 18 days (Fig. 4b). Consistent with TNFR1 driving embryonic lethality owing to RIPK1(K376R), E12.5 *Ripk1*^*K376R/K376R*^ *Tnfr1*^*−/−*^ embryos had normal vascularization (Fig. 4c) and lacked markers of aberrant cell death (Fig. 4c–e and S4C-E). Histological examination of *Ripk1*^*K376R/K376R*^ *Tnfr1*^*−/−*^ pups showed inflammation in several organs, most prominently in the skin (Fig. 4f). These mice also had increased inflammation in the liver, mediastinum, and peritoneum with extension into the pancreas and large intestinal wall. On the other hand, littermate controls had mild to moderate enterocolitis and 3/5 had mild dermatitis. (Figs. 4f and S4F). Levels of several proinflammatory cytokines and chemokines (RANTES, IL-6, MIP-1β, and MCP-1) were also increased in *Ripk1*^*K376R/K376R*^ *Tnfr1*^*−/−*^ pups compared to littermate controls (Fig. 4g). In contrast to TNFR1 deficiency, the combined loss of caspase-8 and RIPK3 produced RIPK1 K376R mice that were viable into adulthood (Fig. S4G). As expected, *Ripk1*^*K376R/K376R*^ *Casp8*^*−/−*^ *Ripk3*^*−/−*^ and *Ripk1*^*+/+*^ *Casp8*^*−/−*^ *Ripk3*^*−/−*^ mice developed the lymphadenopathy that is associated with caspase-8 deficiency [42, 43] (Fig. S4H) and therefore were not aged beyond 11–13 weeks. Interestingly, spleens of *Ripk1*^*K376R/K376R*^ *Casp8*^*−/−*^ *Ripk3*^*−/−*^ mice had a distinct paucity of small lymphocytes (Fig. S4H). Additionally, *Ripk1*^*K376R/K376R*^ *Casp8*^*−/−*^ *Ripk3*^*−/−*^ mice showed increased incidence of perivascular and peribronchiolar lymphoid aggregates in the lung (Fig. 4h).

### RIPK1(K376R) sensitizes adult mice and cells to TNF-induced SIRS and necroptosis

**Fig. 5:**
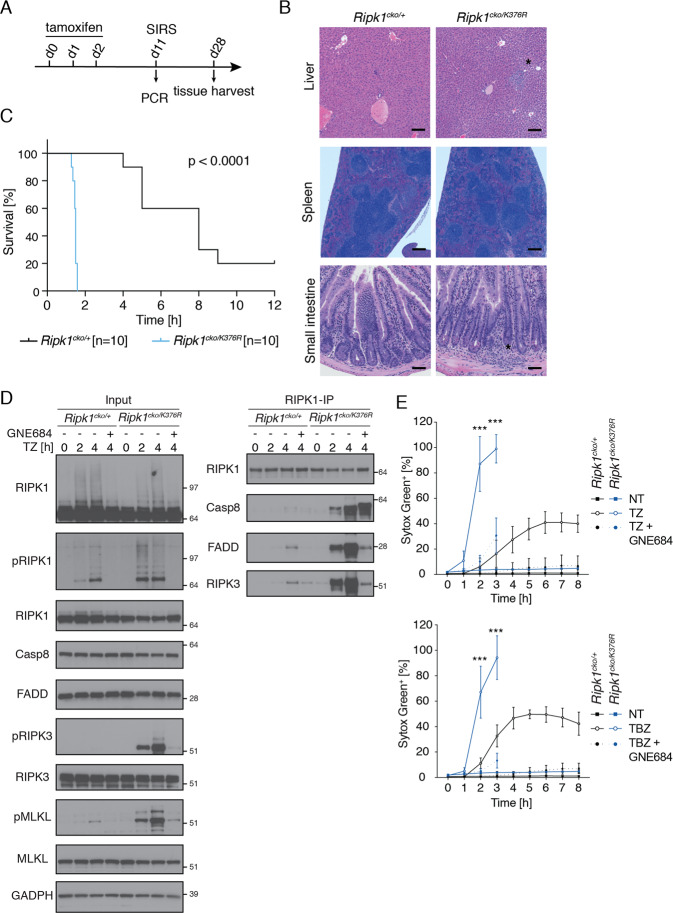
Adult *Ripk1*^*cko/K376R*^ mice are hypersensitive to TNF-induced death. **a** Dosing scheme for deleting the WT allele in *Ripk1*^*K376R/cko*^ *Rosa26-Cre.ER*^*T2*^ or *Ripk1*^*wt/cko*^ *Rosa26-Cre.ER*^*T2*^ animals by tamoxifen [80 mg/kg, IP dosing]. **b** Histology of liver, spleen, and small intestines after 28 days of dosing to induce recombination [scale bar 100 μm liver, 50 μm small intestine, 200 μm spleen]. **c** Kaplan–Meier survival curve of mice after TNF [500 μg/kg iv injections] induced SIRS model at d11 after induced deletion. Difference between the two groups by Mantel–Cox test: *P* < 0.0001. **d** *Ripk1*^*cko/+*^ and *Ripk1*^*cko/K376R*^ BMDMs, treated in vitro with 4-OH-tamoxifen [30 nM] for 5 days, were treated with TNF [100 ng/ml], zVAD [20 μM] and GNE684 [5 μM]. RIPK1-IP was performed and analyzed by western blot for complex II formation. **e** Percentage Sytox Green positive BMDMs, indicating membrane permeabilization and cell death, detected over 8 h by Incucyte with the indicated treatments [TNF – T [5 ng/ml], BV6 – B [500 nM], zVAD – Z [20 μM] and GNE684 [5 μM]. The mean of three independent experiments with SD is plotted. Asterisks indicate statistical analysis between treated *Ripk1*^*cko/+*^ and *Ripk1*^*cko/K376R*^ samples. *P* values, repeated measures two-way ANOVA followed by Tukey’s multiple comparison test (*** > 0.0002).

To study the role of RIPK1 K376 ubiquitination in adult mice, the *Ripk1*^*K376R*^ allele was combined with a conditional *Ripk1* KO allele and a *Rosa26-Cre.ER*^*T2*^ allele for tamoxifen-inducible deletion of the conditional allele. Tamoxifen-treated adult *Rosa26-Cre.ER*^*T2*^ *Ripk1*^*cko/K376R*^ (K376R/-) and *Rosa26-Cre.ER*^*T2*^ w*Ripk1*^*cko/+*^ (WT/-) mice neither lost weight nor showed other signs of illness (Fig. 5a), despite efficient recombination of the conditional KO allele in most tissues by PCR (Fig. S5B). The exceptions were lung, heart, and skin, which showed partial recombination. After 28 days, some of the K376R/- mice exhibited mild inflammation in the liver (2/4 animals) and small intestine (1/4 animals), while all K376R/- mice showed signs of increased hematopoiesis in the spleen (Fig. 5b). Congruent with this mild inflammatory phenotype, levels of TNF and IL-6 in the serum were slightly increased in K376/- mice when compared with WT/- mice (Fig. S5C).

Next, we challenged WT/- and K376R/- animals with TNF. K376R/- mice rapidly succumbed at around 1.5 h, whereas all WT/- mice survived at least 4 h (Fig. 5c). Allele conversion in this experiment was confirmed by PCR for the small and large intestines (Fig. S5D). To further parse out changes in TNF-induced signaling, K376R/- and WT/- BMDMs treated with 4-hydroxytamoxifen during differentiation in vitro were treated with TZ. K376R/- BMDMs showed more robust phosphorylation of RIPK1, RIPK3, and MLKL and increased complex II formation when compared to WT/- BMDMs (Fig. 5d). Accordingly, TZ-treated K376R/- BMDMs died much faster than WT/- BMDMs, and, as described earlier, addition of BV6 did not further enhance K376R/- cell death (Fig. 5e). Treatment with TNF alone also resulted in more prominent cell death signaling in K376R/- BMDMs when compared to WT/- BMDMs (Fig. S5E, F). RIPK1 inhibition with GNE684 blocked all cell death signaling in K376R/- cells (Figs. 5d, e and S5E, F).

### RIPK1(K376R) alters TNFR1 complex formation and reduces K11, K63 and linear RIPK1 ubiquitination

**Fig. 6:**
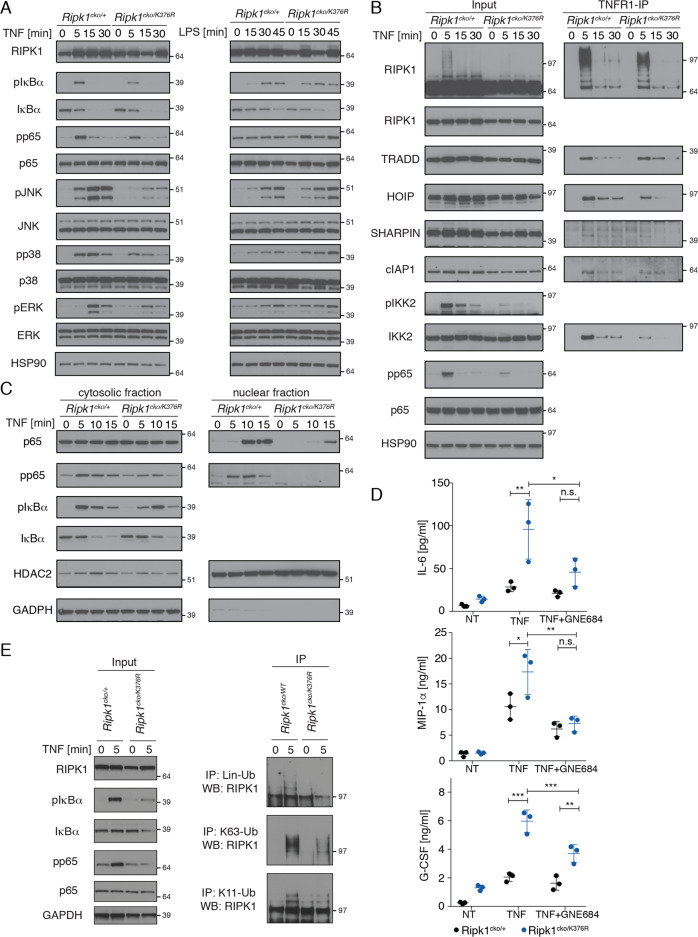
RIPK1 K376R mutation alters TNFR1 complex formation and reduces K11, K63, and linear RIPK1 ubiquitination. *Ripk1*^*cko/+*^ and *Ripk1*^*cko/K376R*^ BMDMs were treated in vitro with 4-OH-tamoxifen [30 nM] for 5 days for all blots shown. **a** Cells were treated with TNF [100 ng/ml] or LPS [10 ng/ml] for indicated times. Cell lysates were analyzed for NF-κB and MAPK activation by western blot. **b** TNFR1-IP of *Ripk1*^*cko/+*^ and *Ripk1*^*cko/K376R*^ BMDMs after TNF [1 μg/ml] treatment for the indicated times. Cell lysates and IPs were analyzed by western blot. **c** Subcellular fractionation of BMDMs treated with 1 μg/ml of TNF for indicated times. Analysis of cytosolic and nuclear fraction. Western blots have the same exposure times and protein amount loaded for the same proteins. **d** Cytokine concentrations in supernatant of BMDMs treated for 8 h with TNF [100 ng/ml] +/− GNE684 [5 μM]. The mean of three independent experiments with SD is plotted. **e** *Ripk1*^*cko/+*^ and *Ripk1*^*cko/K376R*^ BMDMs were treated for 5 min. Cells were lysed in 6 M urea buffer, immunoprecipitated using K11, K63 or linear linkage-specific anti-ubiquitin antibodies and analyzed by western blotting. *P* values, two-way ANOVA followed by Tukey’s multiple comparison test (n.s. not significant, * > 0.0332, ** > 0.0021, *** > 0.0002).

As we observed in K376R MEFs, TNF-induced NF-κΒ and MAPK signaling was impaired in K376R/- BMDMs (Fig. 6a). However, LPS-induced NF-κΒ and MAPK signaling was comparable between K376R/- and WT/- cells, indicating that only the TNFR1 proximal signaling machinery was compromised by RIPK1(K376R) (Fig. 6a). Following TNF treatment, complex I formation was analyzed by TNFR1 IP (Fig. 6b). RIPK1(K376R) did not prevent the ubiquitination-independent recruitment of TRADD and c-IAP1 (Fig. 6b). However, ubiquitination of RIPK1 was reduced in K376R/- cells as well as ubiquitin-dependent recruitment of HOIP, SHARPIN, and IKK2 (Fig. 6b). The same effects were observed by FLAG-IP after FLAG-TNF treatment (Fig. S6A). Besides reduced TNFR1 complex formation, RelA/p65 translocation into the nucleus was also reduced in K376R/- cells (Figs. 6c and S6B). Nonetheless, K376R/- BMDMs released more IL-6, G-CSF, and MIP1α in response to TNF than WT/- BMDMs (Fig. 6d). Inhibition of RIPK1(K376R) with GNE684 significantly reduced cytokine and chemokine secretion, indicating that cell death may play an important role in stimulating cytokine and chemokine secretion in surrounding non-dying cells (Fig. 6d).

Given that K376R cells exhibited decreased TNF-induced RIPK1 ubiquitination and complex I recruitment, we used ubiquitin linkage-specific antibodies to determine which ubiquitin chain linkages were reduced on RIPK1(K376R). Surprisingly, there was an overall decrease in K11-linked, K63-linked, and linear ubiquitination on RIPK1(K376R) when compared with WT RIPK1 (Figs. 6e and S6C). These findings suggest that K376 of RIPK1 could be a site for ubiquitination by multiple linkages, all of them likely contributing to TNF stimulated NF-κB and MAPK signaling as well as complex I stability.

## Discussion

RIPK1 ubiquitination plays a critical role in the spatial and temporal regulation of TNF stimulated inflammatory and cell death signaling [11]. We analyzed the physiological relevance of two described ubiquitination sites on RIPK1, K115, and K376 (ref. [12, 14–17]). Mice expressing only RIPK1(K376R) died during embryonic development around E12.5 with massive cell death in the embryo and yolk sac, as well as upregulation of proinflammatory cytokines and chemokines. Inhibition of RIPK1(K376R) by GNE684 prolonged embryo survival, as did genetic deletion of TNFR1. However, only deletion of caspase-8 and RIPK3 enabled animals to reach adulthood. Together, these data indicate that the absence of K376 RIPK1 ubiquitination causes lethality requiring the kinase activity of RIPK1, TNFR1, and caspase-8/RIPK3 mediated cell death. Consistent with these findings, K376R MEFs and K376R/- BMDMs showed increased sensitivity to TNF and LPS stimulated cell death, while K376R/- mice succumbed rapidly to TNF-induced hypothermia.

Given that lysine 376 of RIPK1 is ubiquitinated within minutes of TNF binding to TNFR1 [12, 13], absence of this site could affect RIPK1 ubiquitination within complex I and subsequent NF-κB and MAPK activation. Indeed, stimulation of RIPK1 K376R cells with TNF lead to reduced RIPK1 ubiquitination with K11-linked, K63-linked and linear ubiquitin chains. This finding may indicate that K376 could be modified by branched chains of different linkages. However, K376 might also be critical for proper localization of RIPK1 within complex I and thus affect modification at other ubiquitination sites. K63-linked ubiquitin chains mediate LUBAC recruitment to TNFR1 associated complex I [20], so recruitment of LUBAC components HOIP and SHARPIN was also sharply decreased in K376R cells. LUBAC deficiency in complex I reduced linear ubiquitination of RIPK1, and this culminated in reduced NF-κB and MAPK signaling, and destabilization of complex I. Although there was reduced expression of NF-κB target genes in K376R MEFs, levels of inflammatory cytokines were higher in K376R/- BMDMs and in K376R embryonic livers. It is possible that cell death releases DAMPs (danger-associated molecular patterns), thus stimulating inflammatory signaling in cells still alive. Taken together, these data suggest that complex I destabilization leading to cell death is the major driver of the RIPK1 K376R phenotype.

Interestingly, BV6 did not increase TNF-induced cell death in K376R cells, which is consistent with deletion of the RIPK1 E3 ligases c-IAP1/2 mainly impacting ubiquitination on K376 of RIPK1. Accordingly, wild type cells with K376 available for ubiquitination were sensitized to TNF-induced death by BV6. These data reiterate the importance of c-IAP1/2 mediated RIPK1 ubiquitination for TNFR1 complex stability. Recently, two other studies reported a similar phenotype of RIPK1 K376R knockin mice [44, 45]. However, those reports focused exclusively on K63-linked RIPK1 ubiquitination, while we found that K11-linked and linear RIPK1 ubiquitination were also severely impacted. Reduced LUBAC recruitment and linear RIPK1 ubiquitination are especially relevant, considering the critical importance of this modification for the stability of complex I and for preventing cell death [20, 46]. We also explored elimination of the K376 RIPK1 ubiquitination site in adult mice and found that this site was not critical for adult homeostasis, at least in the short-term. Still, mice with compromised ubiquitination at K376 of RIPK1 were exquisitely sensitive to TNF-induced hypothermia, arguing that proper TNF signaling requires K376 RIPK1 ubiquitination.

We also characterized the role of K115 RIPK1 ubiquitination [15, 17] and found that elimination of this site mildly sensitized mice or cells to TNF. TNF-induced ubiquitination and signaling in RIPK1 K115R cells appeared normal, likely because RIPK1 undergoes ubiquitination at multiple sites during proinflammatory and cell death signaling [15]. Even though abolition of the K376 ubiquitination site did not completely prevent RIPK1 ubiquitination, it did promote the transition of RIPK1 from complex I to the cell death- promoting complex II. The K115 site evidently does not have such importance and its elimination moderately affects TNF-induced cell death. Further studies on additional RIPK1 ubiquitination sites will be needed to fully explore cell death-associated RIPK1 ubiquitination. Given that therapeutic targeting of RIPK1 is being tested in multiple clinical trials [34, 47], understanding how ubiquitination and other mechanisms regulate activation of RIPK1 may help identify human diseases that would benefit from RIPK1 inhibition.

## Supplementary information

Supplemental Figure Legends

Supplemental Figures

## References

[1] Newton K (2015). RIPK1 and RIPK3: critical regulators of inflammation and cell death. Trends Cell Biol.

[2] Silke J, Rickard JA, Gerlic M (2015). The diverse role of RIP kinases in necroptosis and inflammation. Nat Immunol.

[3] Atretkhany KN, Gogoleva VS, Drutskaya MS, Nedospasov SA (2020). Distinct modes of TNF signaling through its two receptors in health and disease. J Leukoc Biol..

[4] Dondelinger Y, Delanghe T, Priem D, Wynosky-Dolfi MA, Sorobetea D, Rojas-Rivera D (2019). Serine 25 phosphorylation inhibits RIPK1 kinase-dependent cell death in models of infection and inflammation. Nat Commun..

[5] Geng J, Ito Y, Shi L, Amin P, Chu J, Ouchida AT (2017). Regulation of RIPK1 activation by TAK1-mediated phosphorylation dictates apoptosis and necroptosis. Nat Commun..

[6] Degterev A, Huang Z, Boyce M, Li Y, Jagtap P, Mizushima N (2005). Chemical inhibitor of nonapoptotic cell death with therapeutic potential for ischemic brain injury. Nat Chem Biol.

[7] Degterev A, Hitomi J, Germscheid M, Ch’en IL, Korkina O, Teng X (2008). Identification of RIP1 kinase as a specific cellular target of necrostatins. Nat Chem Biol.

[8] Newton K, Wickliffe KE, Dugger DL, Maltzman A, Roose-Girma M, Dohse M (2019). Cleavage of RIPK1 by caspase-8 is crucial for limiting apoptosis and necroptosis. Nature..

[9] Lalaoui N, Boyden SE, Oda H, Wood GM, Stone DL, Chau D (2020). Mutations that prevent caspase cleavage of RIPK1 cause autoinflammatory disease. Nature..

[10] Tao P, Sun J, Wu Z, Wang S, Wang J, Li W (2019). A dominant autoinflammatory disease caused by non-cleavable variants of RIPK1. Nature..

[11] Witt A, Vucic D (2017). Diverse ubiquitin linkages regulate RIP kinases-mediated inflammatory and cell death signaling. Cell Death Differ.

[12] Ea CK, Deng L, Xia ZP, Pineda G, Chen ZJ (2006). Activation of IKK by TNFalpha requires site-specific ubiquitination of RIP1 and polyubiquitin binding by NEMO. Mol Cell.

[13] Li H, Kobayashi M, Blonska M, You Y, Lin X (2006). Ubiquitination of RIP is required for tumor necrosis factor α-induced NF-κB activation. J Biol Chem.

[14] Li H, Kobayashi M, Blonska M, You Y, Lin X (2006). Ubiquitination of RIP is required for tumor necrosis factor alpha-induced NF-kappaB activation. J Biol Chem.

[15] de Almagro MC, Goncharov T, Izrael-Tomasevic A, Duttler S, Kist M, Varfolomeev E (2017). Coordinated ubiquitination and phosphorylation of RIP1 regulates necroptotic cell death. Cell Death Differ.

[16] Amin P, Florez M, Najafov A, Pan H, Geng J, Ofengeim D (2018). Regulation of a distinct activated RIPK1 intermediate bridging complex I and complex II in TNFalpha-mediated apoptosis. Proc Natl Acad Sci USA.

[17] Wang H, Meng H, Li X, Zhu K, Dong K, Mookhtiar AK (2017). PELI1 functions as a dual modulator of necroptosis and apoptosis by regulating ubiquitination of RIPK1 and mRNA levels of c-FLIP. Proc Natl Acad Sci USA.

[18] Varfolomeev E, Goncharov T, Fedorova AV, Dynek JN, Zobel K, Deshayes K (2008). c-IAP1 and c-IAP2 are critical mediators of tumor necrosis factor alpha (TNFα)-induced NF-κB activation. J Biol Chem.

[19] Dynek JN, Goncharov T, Dueber EC, Fedorova AV, Izrael-Tomasevic A, Phu L (2010). c-IAP1 and UbcH5 promote K11-linked polyubiquitination of RIP1 in TNF signalling. Embo J.

[20] Bertrand MJ, Milutinovic S, Dickson KM, Ho WC, Boudreault A, Durkin J (2008). cIAP1 and cIAP2 facilitate cancer cell survival by functioning as E3 ligases that promote RIP1 ubiquitination. Mol Cell..

[21] Annibaldi A, Wicky John S, Vanden Berghe T, Swatek KN, Ruan J, Liccardi G (2018). Ubiquitin-mediated regulation of RIPK1 kinase activity independent of IKK and MK2. Mol Cell.

[22] Haas TL, Emmerich CH, Gerlach B, Schmukle AC, Cordier SM, Rieser E (2009). Recruitment of the linear ubiquitin chain assembly complex stabilizes the TNF-R1 signaling complex and is required for TNF-mediated gene induction. Mol Cell..

[23] Kondylis V, Kumari S, Vlantis K, Pasparakis M (2017). The interplay of IKK, NF-kappaB and RIPK1 signaling in the regulation of cell death, tissue homeostasis and inflammation. Immunol Rev..

[24] Shim JH, Xiao C, Paschal AE, Bailey ST, Rao P, Hayden MS (2005). TAK1, but not TAB1 or TAB2, plays an essential role in multiple signaling pathways in vivo. Genes Dev.

[25] Sabio G, Davis RJ (2014). TNF and MAP kinase signalling pathways. Semin Immunol.

[26] Holbrook J, Lara-Reyna S, Jarosz-Griffiths H, McDermott M (2019). Tumour necrosis factor signalling in health and disease. F1000Res.

[27] Murphy JM, Czabotar PE, Hildebrand JM, Lucet IS, Zhang JG, Alvarez-Diaz S (2013). The pseudokinase MLKL mediates necroptosis via a molecular switch mechanism. Immunity..

[28] Sun L, Wang H, Wang Z, He S, Chen S, Liao D (2012). Mixed lineage kinase domain-like protein mediates necrosis signaling downstream of RIP3 kinase. Cell..

[29] Wang H, Sun L, Su L, Rizo J, Liu L, Wang L-F (2014). Mixed lineage kinase domain-like protein MLKL causes necrotic membrane disruption upon phosphorylation by RIP3. Mol Cell.

[30] Vanden Berghe T, Linkermann A, Jouan-Lanhouet S, Walczak H, Vandenabeele P (2014). Regulated necrosis: the expanding network of non-apoptotic cell death pathways. Nat Rev Mol Cell Biol.

[31] Patel S, Webster JD, Varfolomeev E, Kwon YC, Cheng JH, Zhang J (2020). RIP1 inhibition blocks inflammatory diseases but not tumor growth or metastases. Cell Death Differ.

[32] Yuan J, Amin P, Ofengeim D (2019). Necroptosis and RIPK1-mediated neuroinflammation in CNS diseases. Nat Rev Neurosci.

[33] Newton K, Dugger DL, Wickliffe KE, Kapoor N, de Almagro MC, Vucic D (2014). Activity of protein kinase RIPK3 determines whether cells die by necroptosis or apoptosis. Science.

[34] Martens S, Hofmans S, Declercq W, Augustyns K, Vandenabeele P (2020). Inhibitors targeting RIPK1/RIPK3: old and new drugs. Trends Pharm Sci.

[35] Peschon JJ, Torrance DS, Stocking KL, Glaccum MB, Otten C, Willis CR (1998). TNF receptor-deficient mice reveal divergent roles for p55 and p75 in several models of inflammation. J Immunol.

[36] Seibler J, Zevnik B, Kuter-Luks B, Andreas S, Kern H, Hennek T (2003). Rapid generation of inducible mouse mutants. Nucleic Acids Res.

[37] Cong L, Ran FA, Cox D, Lin S, Barretto R, Habib N (2013). Multiplex genome engineering using CRISPR/Cas systems. Science.

[38] Mali P, Yang L, Esvelt KM, Aach J, Guell M, DiCarlo JE (2013). RNA-guided human genome engineering via Cas9. Science..

[39] Pennica D, Hayflick JS, Bringman TS, Palladino MA, Goeddel DV (1985). Cloning and expression in Escherichia coli of the cDNA for murine tumor necrosis factor. Proc Natl Acad Sci USA.

[40] Varfolomeev E, Blankenship JW, Wayson SM, Fedorova AV, Kayagaki N, Garg P (2007). IAP antagonists induce autoubiquitination of c-IAPs, NF-κB activation, and TNFα-dependent apoptosis. Cell..

[41] de Almagro MC, Goncharov T, Newton K, Vucic D (2015). Cellular IAP proteins and LUBAC differentially regulate necrosome-associated RIP1 ubiquitination. Cell Death Dis.

[42] Kaiser WJ, Upton JW, Long AB, Livingston-Rosanoff D, Daley-Bauer LP, Hakem R (2011). RIP3 mediates the embryonic lethality of caspase-8-deficient mice. Nature.

[43] Oberst A, Dillon CP, Weinlich R, McCormick LL, Fitzgerald P, Pop C (2011). Catalytic activity of the caspase-8-FLIP(L) complex inhibits RIPK3-dependent necrosis. Nature..

[44] Tang Y, Tu H, Zhang J, Zhao X, Wang Y, Qin J (2019). K63-linked ubiquitination regulates RIPK1 kinase activity to prevent cell death during embryogenesis and inflammation. Nat Commun..

[45] Zhang X, Zhang H, Xu C, Li X, Li M, Wu X (2019). Ubiquitination of RIPK1 suppresses programmed cell death by regulating RIPK1 kinase activation during embryogenesis. Nat Commun..

[46] Peltzer N, Walczak H (2019). Cell death and inflammation – a vital but dangerous liaison. Trends Immunol.

[47] Jensen S, Seidelin JB, LaCasse ES, Nielsen OH (2020). SMAC mimetics and RIPK inhibitors as therapeutics for chronic inflammatory diseases. Sci Signal.

