# Supplementary material for: Impaired RIPK1 ubiquitination sensitizes mice to TNF toxicity and inflammatory cell death
Source: Cell Death Differ. 2020 Sep 30;28(3):985–1000. doi: 10.1038/s41418-020-00629-3 (PMC7937686; doi:10.1038/s41418-020-00629-3)
Supplement: Supplementary file 1 — Supplemental Figure Legends [file 41418_2020_629_MOESM1_ESM.docx]

**Impaired RIPK1 ubiquitination sensitizes mice to TNF toxicity and inflammatory cell death**

Kist et al.

**Supplementary Figures S1-S6**

**Supplementary Figure 1.** **RIPK1 K376R mutation results in embryonic lethality, which can be delayed by RIPK1 kinase inhibition with GNE684.**

**Supplementary Figure 2.** **RIPK1 K115R mutation does not affect TNF induced NF-κB and MAPK signaling but sensitizes to cell death.**

**Supplementary Figure 3. *Ripk1^K376R/K376R^* affects TNF induced gene expression and promotes cell death via different stimuli.**

**Supplementary Figure 4. LPS induced cell death in *Ripk1^K376R/K376R^* MEFs. *Tnfr1^-/-^* delays lethality to after birth, while cross to *Casp8^-/-^Ripk3^-/-^* mice allow survival to adulthood.**

**Supplementary Figure 5. Inducible deletion of *Ripk1* wt allele in *Ripk1^cko/K376R^* knockin mice.**

**Supplementary Figure 6.** **Diminished TNF stimulated NF-κB activation and linear ubiquitination in *Ripk1^cko/K376R^* BMDMs.**

**Supplementary Figure 1.** **RIPK1 K376R mutation results in embryonic lethality, which can be delayed by RIPK1 kinase inhibition with GNE684**. (A) Representative images of *Ripk1^+/+^* and *Ripk1^K376R/K376R^* embryos with and without yolk sac and placenta for E10.5 to E13.5. Scale bars on the top left of each image indicate 2 mm. (B) IHC for cleaved caspase-3 of *Ripk1^+/+^* and *Ripk1^K376R/K376R^* E11.5 embryos. [scale bar 200 μm] (C) IHC for pRIPK3 in the placenta labyrinths of *Ripk1^+/+^* and *Ripk1^K376R/K376R^* embryos at E11.5 and E12.5 [scale bar 50 μm]. (D) Quantification of IHC for pRIPK3 positive cells in placenta labyrinths at E11.5 and E12.5 in *Ripk1^+/+^* and *Ripk1^K376R/K376R^* (n=3). (E) IHC for cleaved caspase-3 of the representative placentas of *Ripk1^K376R/+^* [scale bar 100 μm]. (F) IHC for pRIPK3 of the placenta of *Ripk1^+/+^*, *Ripk1^K376R/+^* and *Ripk1^K376R/K376R^* embryos at E12.5 treated with vehicle or GNE684 [scale bar 100 μm]. (G) RT-qPCR analysis of indicated cytokines and chemokines using E11.5 embryonic liver total RNA [n = 3] after dosing for three days with vehicle or 50 mg/kg GNE684. Data of three independent experiments are shown as mean with SD. P values, one-way ANOVA followed by Tukey’s multiple comparison test (** > 0.0021, *** > 0.0002).

**Supplementary Figure 2.** **RIPK1 K115R mutation does not affect TNF induced NF-κB and MAPK signaling but sensitizes to cell death.** (A) Temperature blot of *Ripk1^+/+^* in black [n = 13] and *Ripk1^K115R/K115R^* in red [n = 13] mice in TNF induced systemic inflammatory response syndrome [SIRS] with 500 μg/kg TNF injected iv [from Fig. 1g]. (B) Representative cytokines and chemokines were analyzed by Luminex at 4 h after iv TNF injection [500 μg/kg] *Ripk1^+/+^* in black [n = 6] and *Ripk1^K115R/K115R^* in red [n = 5]. (C) *Ripk1^+/+^* and *Ripk1^K115R/K115R^* BMDMs were treated with TNF [1 μg/ml] for the indicated times. Western blot analysis of lysates was performed for pathway activation. (D) *Ripk1^+/+^* and *Ripk1^K115R/K115R^* primary MEFs were treated with TNF [100 ng/ml], zVAD [20 μM] +/- GNE684 [5 μM] for up to 4 h. Cell lysates were by analyzed by Western Blot with the indicated antibodies. (E) *Ripk1^+/+^* and *Ripk1^K115R/K115R^* BMDMs were treated with TNF [100 ng/ml], zVAD [20 μM] +/- GNE684 [5 μM] for up to 4 h. Cells were lysed and immunoprecipitations were performed with RIPK1 antibody. IPs and cell lysates were analyzed by Western blotting with indicated antibodies. (F) *Ripk1^+/+^* and *Ripk1^K115R/K115R^* BMDMs were treated with TNF [100 ng/ml], zVAD [20 μM]. Samples were lysed in 6 M urea buffer and immunoprecipitated using K63 or linear linkage-specific anti-ubiquitin antibodies and analyzed by Western blotting. (G) Percentage Sytox Green positive BMDMs, indicating membrane permeabilization and cell death, detected over 8 h by Incucyte with the indicated treatments [TNF - T [5 ng/ml], BV6 - B [500 nM], zVAD - Z [20 μM] +/- GNE684 [5 μM]] (n = 2 independent for data sets for KI NT and WT TZ, n = 3 independent experiments for all other treatments). For each time point the mean with SD is plotted. Asterixis indicate statistical analysis between treated WT and K115R samples. P values, repeated measures two-way ANOVA followed by Tukey’s multiple comparison test (* > 0.0332, ** > 0.0021, *** > 0.0002).

**Supplementary Figure 3. *Ripk1^K376R/K376R^* affects TNF induced gene expression and promotes cell death via different stimuli.** (A) *Ripk1^+/+^*, *Ripk1^K376R/+^* and *Ripk1^K376R/+^* primary MEFs were treated with TNF [1 μg/ml] for the indicated times and analyzed by Western Blot. (B) Subcellular fractionation of immortalized MEFs treated with 1 μg/ml of TNF for indicated times and analyzed by Western Blot. (C) RT-qPCR for *Ccl2*, *Il1b* and *Tnfaip3* for 2 independent experiments with technical duplicates in primary MEFs treated with 100 ng/ml TNF. The left column is normalized to non-treated WT MEFs and the right column shows normalization of treated samples to WT or K376R non-treated samples. (D) *Ripk1^+/+^*, *Ripk1^K376R/+^* and *Ripk1^K376R^****^/+^*** primary MEFs were treated with TNF [100 ng/ml] and zVAD [20 μM] for the indicated times. Cell lysis was performed in lysis buffer + 1% SDS and the samples were analyzed by Western Blot. (E) Western Blot of primary MEFs after treatment with TNF - T [100 ng/ml], BV6 [1 μM], zVAD - Z [20 μM] and GNE684 [5 μM] for the indicated time points.

**Supplementary Figure 4. *LPS induced cell death in Ripk1^K376R/K376R^ MEFs*. *Tnfr1^-/-^* delays lethality to after birth, while cross to *Casp8^-/-^Ripk3^-/-^* mice allow survival to adulthood.** (A) Percentage of dead primary MEFs [Sytox Green positive cells] compared to positive control. Signal was measured every hour for 20 h in the Incucyte with the indicated treatments: LPS - L [1 μg/ml], zVAD – Z [20 μM], GNE684 [5 μM]. 2 independent experiments in technical quadruplicates are shown. Asterixis indicate statistical analysis between treated WT and K376R samples. After 6h the significance did not change. (B) Western Blot analysis of primary MEFs treated with LPS – L [1 μg/ml], zVAD – Z [20 μM], GNE684 5 μM. P values, repeated measures two-way ANOVA followed by Tukey’s multiple comparison test (* > 0.0332, ** > 0.0021, *** > 0.0002). (C) IHC for pRIPK3 in the placenta labyrinths of *Ripk1^+/+^* and *Ripk1^K376R/K376R^* embryos at E11.5 [scale bar 200 μm]. (D) IHC for pRIPK3 in embryos and placenta of *Ripk1^+/+^* and *Ripk1^K376R/K376R^* embryos [scale bar 200 μm]. (E) Individual pRIPK3 positive cells in a *Ripk1^K376R/K376R^ Tnfr1^-/-^* embryo and yolk sac [scale bar 50 μm]. (F) Representative histology of pancreas and large intestine of *Tnfr1^-/-^ Ripk1^+/+^* and *Tnfr1^-/-^ Ripk1^K376R/K376R^* mice [scale bar 200 μm]. (G) Number of mice generated from three different breedings: Cross of *Ripk1^K376R/+^ Casp8^-/-^ Ripk3^-/-^* to *Ripk1^K376R/+^ Casp8^-/-^ Ripk3^-/-^* or *Ripk1^K376R/+^ Casp8^+/-^ Ripk3^-/-^* and *Ripk1^K376R/K376R^ Casp8^-/-^ Ripk3^-/-^* to *Ripk1^K376R/+^ Casp8^+/-^ Ripk3^-/-^*. (H) Lymph nodes [scale bar 100 μm] and spleen [scale bar 200 μm] of adult *Ripk1^K376R/K376R^ Casp8^-/-^ Ripk3^-/-^* and *Ripk1^+/+^ Casp8^-/-^ Ripk3^-/-^* mice. Lymph nodes of K376R KI animals were expanded and effaced with monomorphic lymphocytes compared to WT. K376R KI spleens showed loss of follicular architecture and paucity of small lymphocytes.

**Supplementary Figure 5. Inducible deletion of *Ripk1* wt allele in *Ripk1^cko/K376R^* knockin mice.** (A) Schematic organization of the *Ripk1* conditional knockout allele. (B) PCR analysis of tamoxifen induced recombination in major organs of male and female *Ripk1^cko/+^* and *Ripk1^cko/K376R^* mice at d11 after first tamoxifen dosing. (C) Serum cytokine levels in *Ripk1^cko/+^* [n = 3] and *Ripk1^cko/K376R^* [n = 4] animals after 28 days of tamoxifen induced deletion. (D) PCR analysis after tamoxifen induced recombination at d11 after dosing. Samples of small and large intestine were collected of all animals from the SIRS study (Fig. 5C) to confirm conversion of the cko-allele. (E) *Ripk1^cko/+^* and *Ripk1^cko/K376R^* BMDMs, in vitro treated with 4-OH-tamoxifen [30 nM] for 5 days, were treated with TNF [100 ng/ml], zVAD [20 μM] and GNE684 [5 μM]. Cell lysates were analyzed by Western Blot for pathway activation. (F) Percentage Sytox Green positive BMDMs, indicating membrane permeabilization and cell death, detected over 8 h by Incucyte with the indicated treatments [TNF - T [5 ng/ml], BV6 - B [500 nM] and GNE684 [5 μM]]. Means with SD are plotted. P values, unpaired two tailed t-test (B) (n.s. = non-significant). Asterixis indicate statistical analysis between treated *Ripk1^cko/+^* and *Ripk1^cko/K376R^* samples. For both treatments the significance did not change after 3 h. P values, repeated measures two-way ANOVA followed by Tukey’s multiple comparison test (** > 0.0021, *** > 0.0002).

**Supplementary Figure 6.** **Diminished TNF stimulated NF-κB activation and linear ubiquitination in *Ripk1^cko/K376R^* BMDMs.** *Ripk1^cko/+^* and *Ripk1^cko/K376R^* BMDMs were treated *in vitro* with 4-OH-tamoxifen [30 nM] for 5 days for all blots shown. (A) *Ripk1^cko/+^* and *Ripk1^cko/K376R^* BMDMs were treated with FLAG-TNF [1 μg/ml] for the indicated times and IP was performed with anti-FLAG M2 magnetic beads. Cell lysates and IPs were analyzed by Western blotting with indicated antibodies. (B) Immunofluorescence of BMDMs treated with TNF for 10 minutes and stained with anti-p65 antibody [red] and DAPI [green] [scale bar 50 μm]. Lower panel shows two-fold enlarged ROI of p65 staining. (C) *Ripk1^cko/+^* and *Ripk1^cko/K376R^* BMDMs were treated for 5 minutes with TNF [100 ng/ml]. Cells were lysed in 6 M urea buffer, immunoprecipitated using linear linkage-specific anti-ubiquitin antibodies and analyzed by Western blotting.
