# Supplementary material for: Impaired RIPK1 ubiquitination sensitizes mice to TNF toxicity and inflammatory cell death
Source: Cell Death Differ. 2020 Sep 30;28(3):985–1000. doi: 10.1038/s41418-020-00629-3 (PMC7937686; doi:10.1038/s41418-020-00629-3)

Supplementary Figure 1

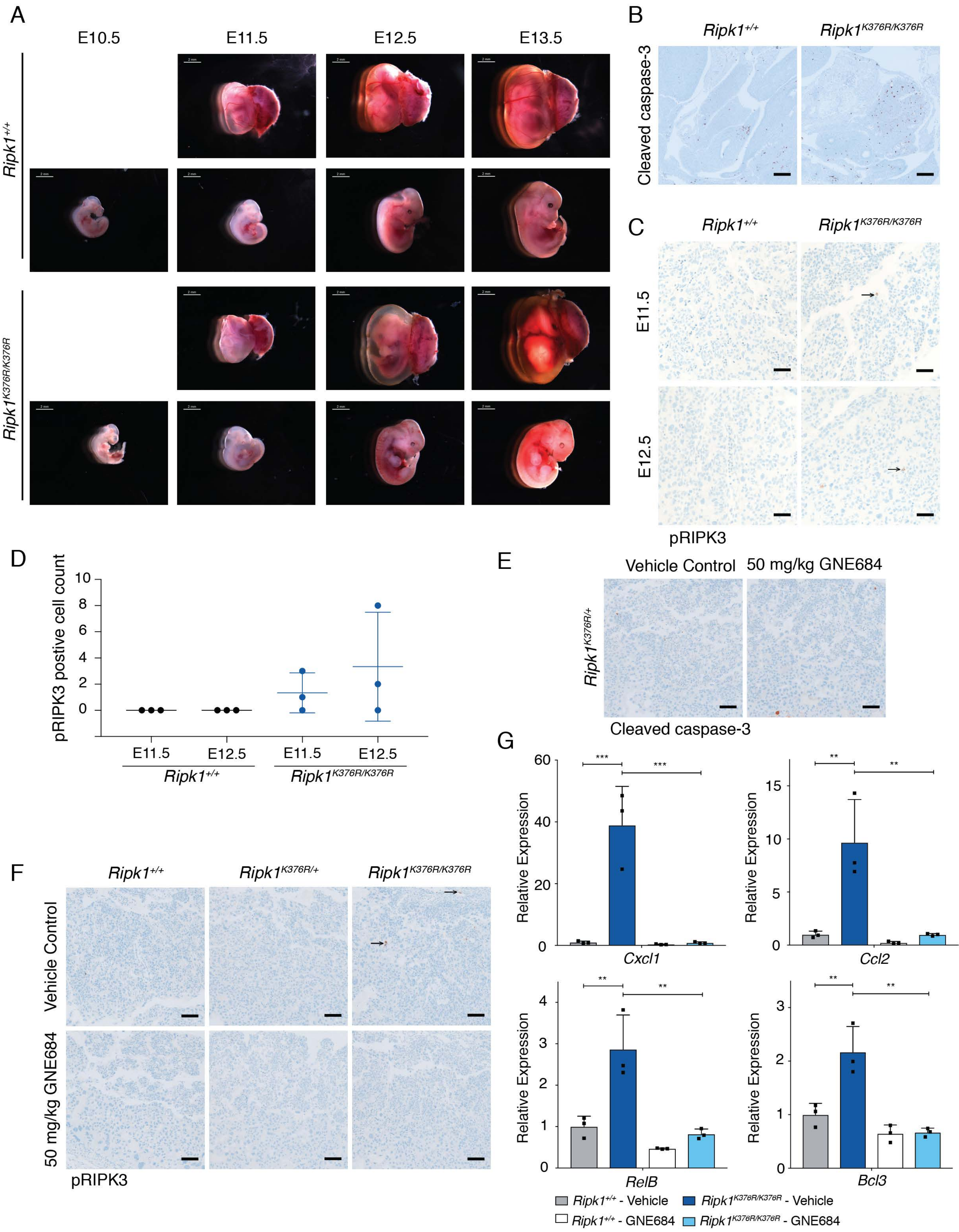

Supplementary Figure 2

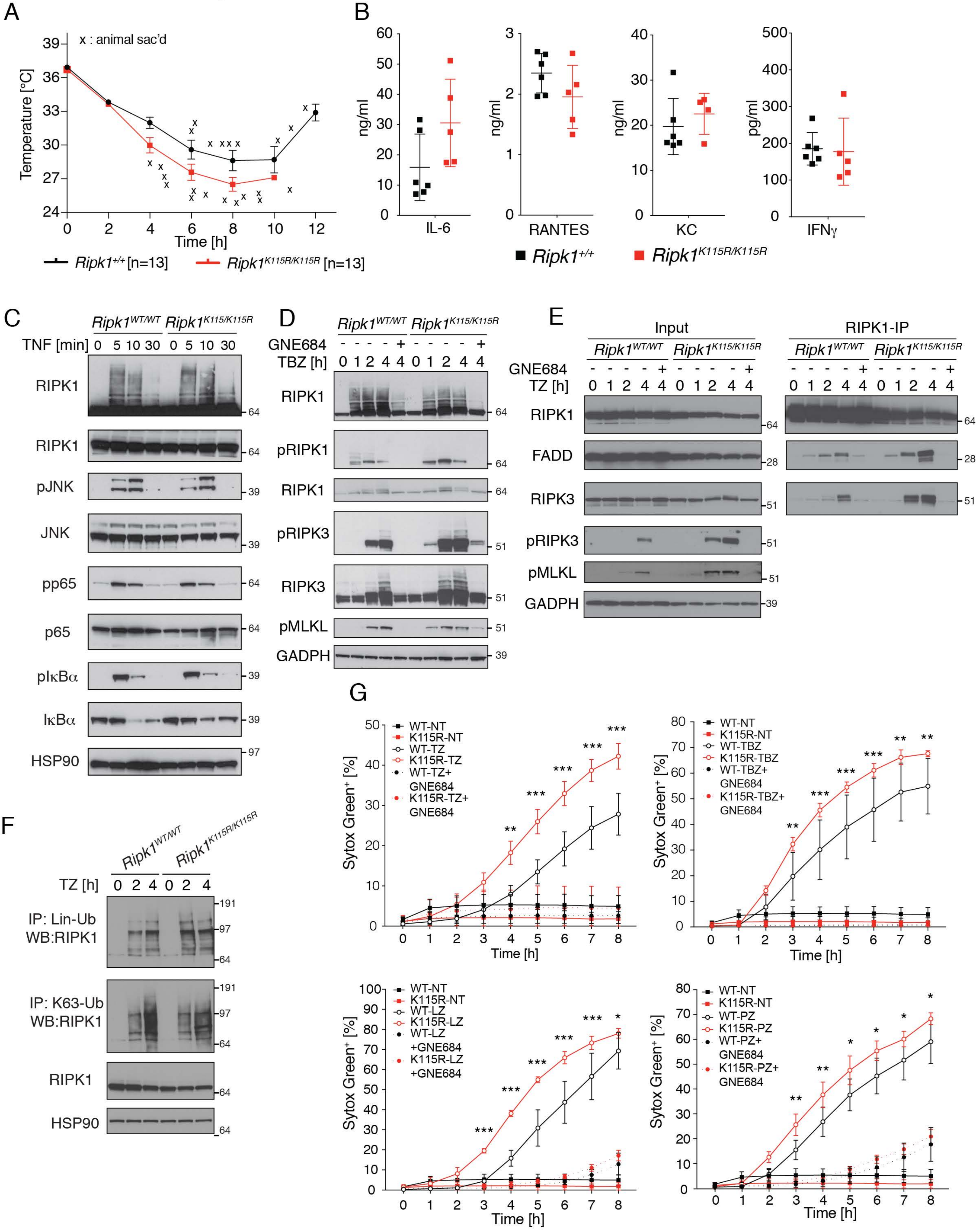

Supplementary Figure 3

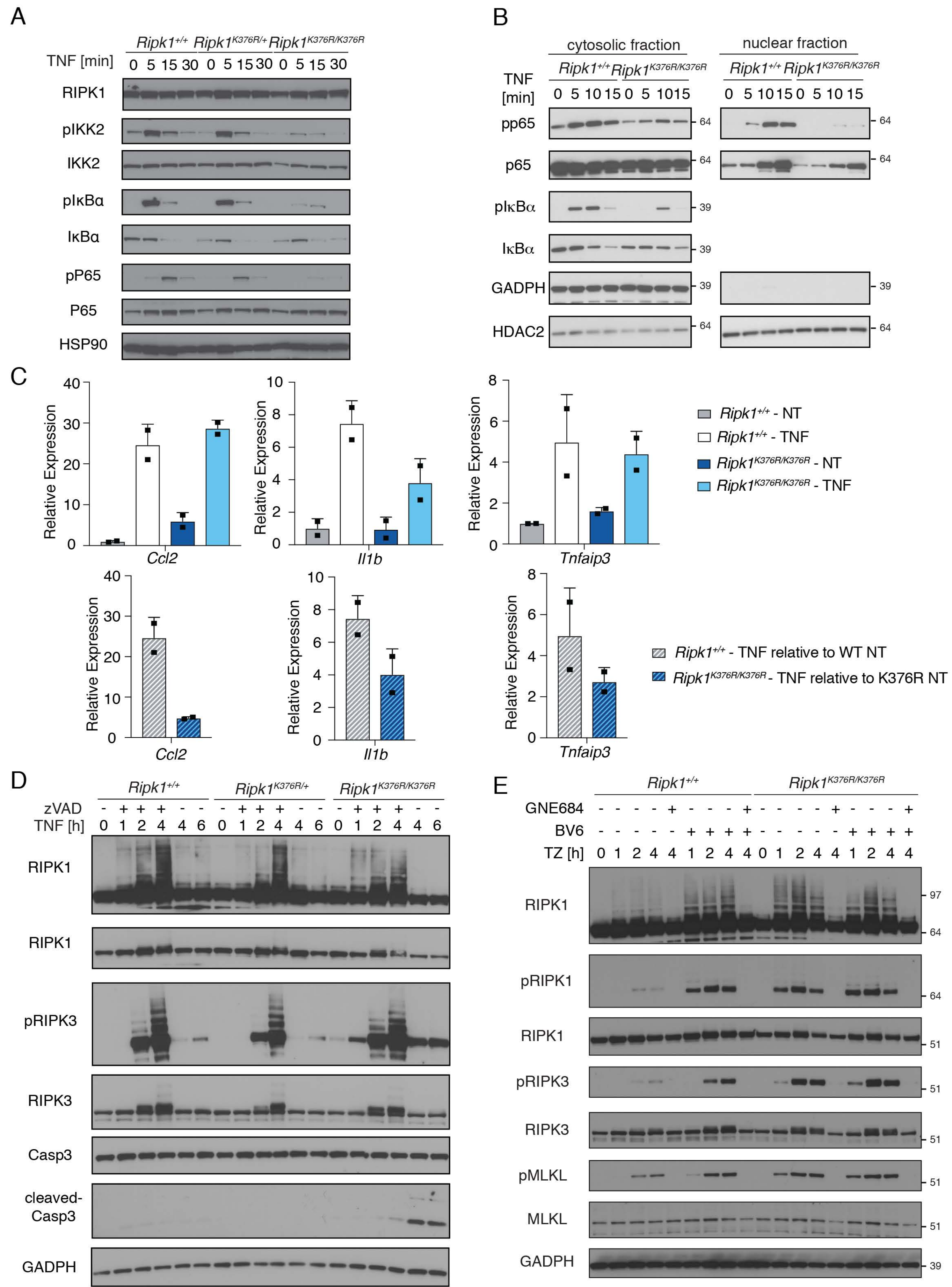

Supplementary Figure 4

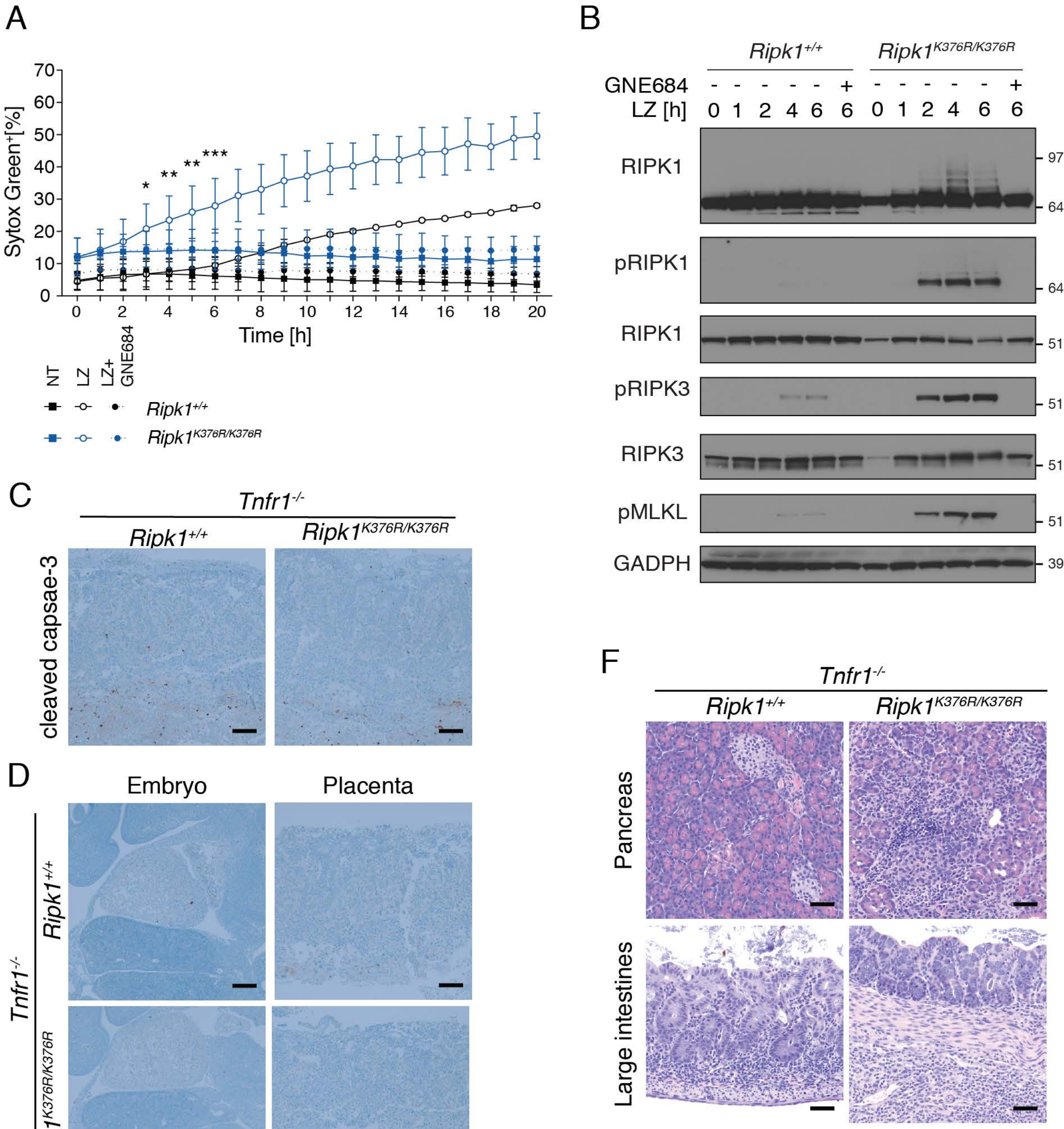

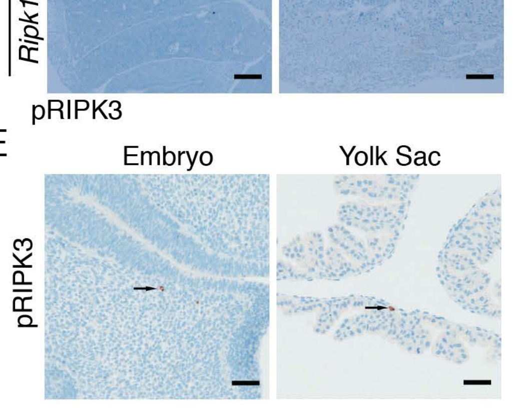

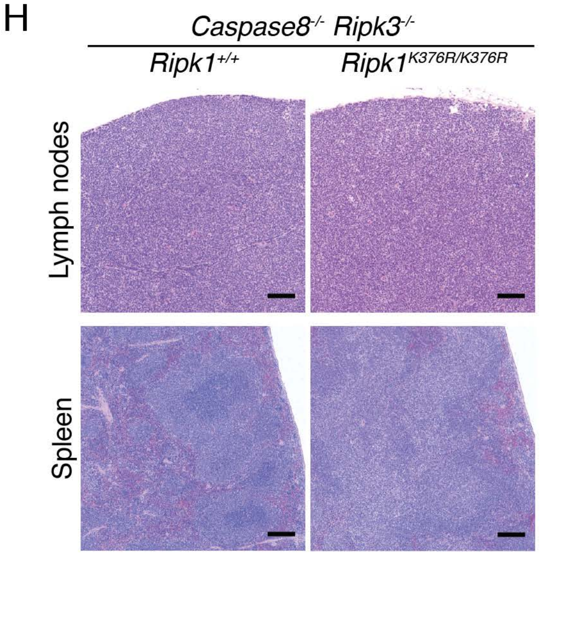

G

	Ripk1$^{+/+}$ *Casp8*$^{-/-}$ *Ripk3*$^{-/-}$	*various genotypes**	*Ripk1*$^{K376R/K376R}$ *Casp8*$^{-/-}$ *Ripk3*$^{-/-}$
Adult	7	17	7

*: 9 *Ripk1*$^{K376R/+}$ *Casp8*$^{-/-}$ *Ripk3*$^{-/-}$
5 *Ripk1*$^{K376R/+}$ *Casp8*$^{+/-}$ *Ripk3*$^{-/-}$
3 *Ripk1*$^{+/+}$ *Casp8*$^{+/-}$ *Ripk3*$^{-/-}$

Supplementary Figure 5

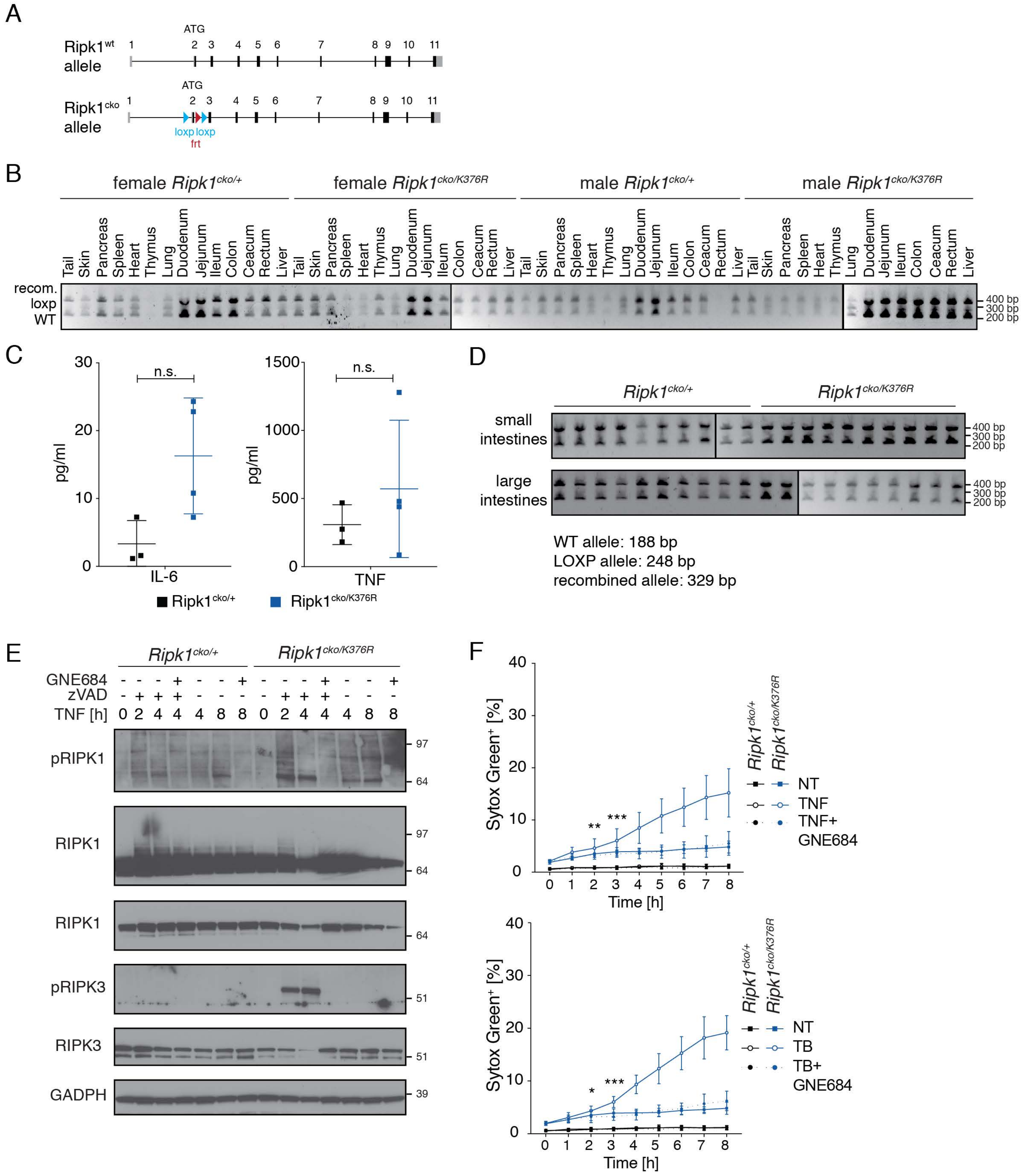

Supplementary Figure 6

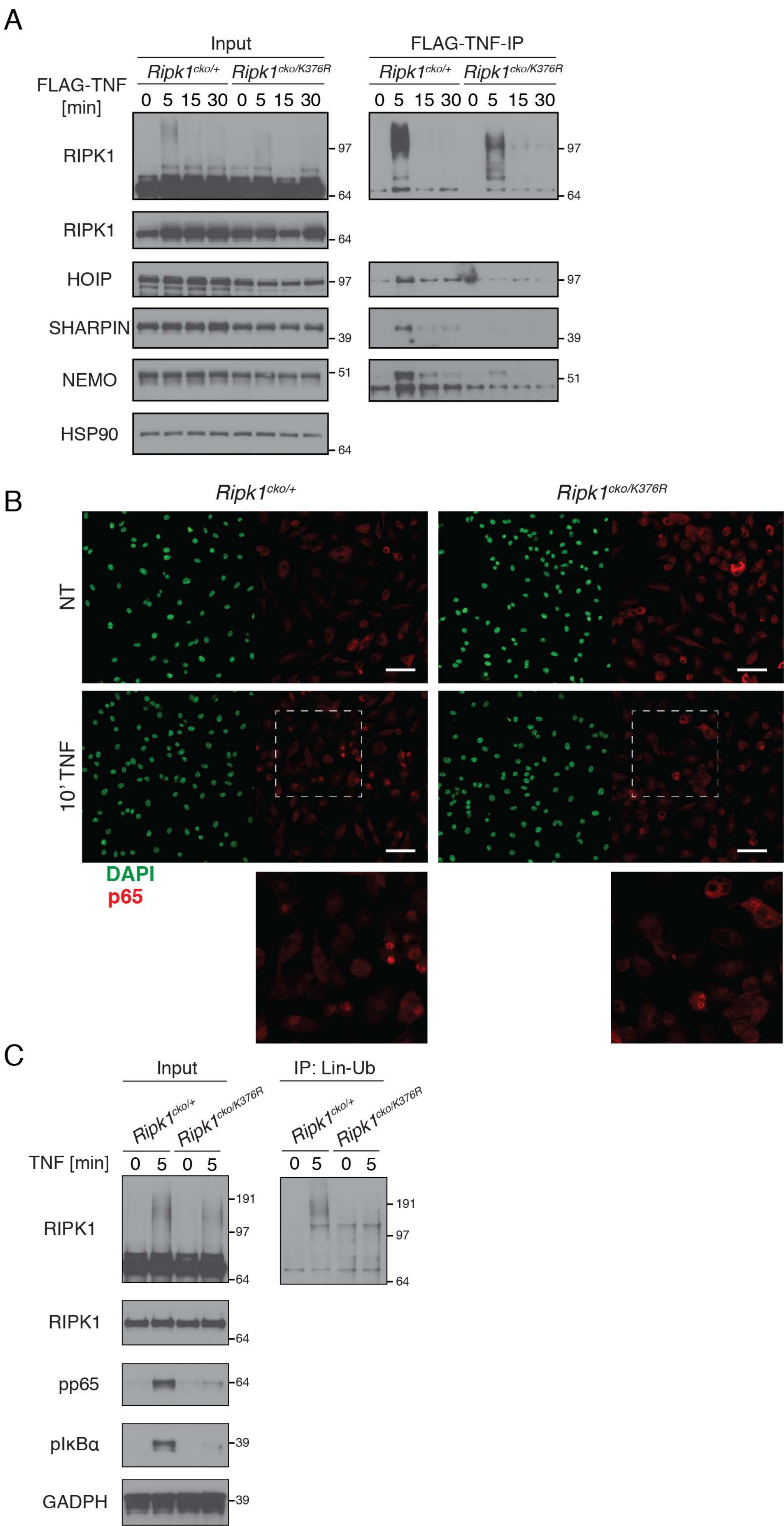

Supplement: Supplementary file 2 — Supplemental Figures [file 41418_2020_629_MOESM2_ESM.pdf]
